# A Simultaneous Determination of the B_1_ and B_6_ Vitamers Reveals Their Loss During a Single Peritoneal Dialysis Session: Chromatographic and Chemometric Approach

**DOI:** 10.3390/ijms26157177

**Published:** 2025-07-25

**Authors:** Paweł Rudnicki-Velasquez, Karol Krzymiński, Magdalena Jankowska, Anna Baraniak, Paulina Czaplewska

**Affiliations:** 1 Department of Falsified Medicines and Medical Devices, National Medicines Institute, 00-725 Warsaw, Poland; 2 Faculty of Chemistry, University of Gdańsk, 80-308 Gdańsk, Poland; karol.krzyminski@ug.edu.pl; 3 Department of Nephrology, Transplantology and Internal Medicine, Medical University of Gdańsk, 80-210 Gdańsk, Poland; magdalena.jankowska@gumed.edu.pl; 4 Department of Pharmaceutical Microbiology and Laboratory Diagnostics, National Medicines Institute, 00-725 Warsaw, Poland; a.baraniak@nil.gov.pl; 5 Laboratory of Mass Spectrometry-Core Facility Laboratories, Intercollegiate Faculty of Biotechnology of UG and MUG, University of Gdańsk, 80-307 Gdańsk, Poland; paulina.czaplewska@ug.edu.pl

**Keywords:** peritoneal dialysis, vitamin B_1_, vitamin B_6_, thiamine diphosphate, pyridoxal, LC-MS/MS, HPLC, chemometrics, vitamin loss, renal replacement therapy

## Abstract

This study aimed to assess the extent of vitamin B_1_ and B_6_ vitamer loss during a single peritoneal dialysis (PD) session using a combination of chromatographic techniques and chemometric analysis. Dialysis effluent samples were collected from 41 PD patients (22 on continuous ambulatory peritoneal dialysis (CAPD) and 19 on automated peritoneal dialysis (APD)) during a standardised peritoneal equilibration test. Concentrations of thiamine monophosphate, thiamine diphosphate (ThDP), pyridoxine, pyridoxal (PL), and pyridoxamine were determined using high-performance liquid chromatography with a fluorescence detector. The analytical method was validated in terms of sensitivity, linearity, accuracy, and recovery. Multiple regression analysis was employed to identify potential clinical and demographic predictors of vitamin washout. All vitamers except pyridoxal 5-phosphate (PLP) were detectable in dialysis effluents. ThDP exhibited the greatest loss among the B_1_ forms (ca. 0.05–0.57 mg/24 h), while PL exhibited the most significant loss among the B_6_ forms (ca. 0.01–0.19 mg/24 h). Vitamin losses varied depending on the dialysis modality (continuous ambulatory peritoneal dialysis, or CAPD, versus automated peritoneal dialysis, or APD) and the peritoneal transport category. Regression analysis identified body weight, haemoglobin, and haematocrit as independent predictors of ThDP washout (R^2^ = 0.58). No statistically robust models were established for the other vitamers. Even short medical procedures (such as single PD) can result in measurable losses of water-soluble vitamins, particularly ThDP and PL. The results emphasise the importance of personalised vitamin supplementation for PD patients and suggest that body composition and haematological parameters significantly influence the loss of thiamine.

## 1. Introduction

Progressive chronic kidney disease (CKD) requires the initiation of renal replacement therapy (RRT), which aims to be a substitute for disordered kidney function. RRT is particularly essential for patients with symptoms of uraemia [1]. Apart from kidney transplantation, which remains the most effective treatment, RRT includes haemodialysis (HD) and peritoneal dialysis (PD) to eliminate uremic solutes and excess fluids from the patient’s body. PD treatment can be divided into continuous ambulatory peritoneal dialysis (CAPD) and automated peritoneal dialysis (APD). CAPD typically involves four 2-litre dwellings of dialysate per day at 4 to 8 h intervals. In contrast, APD automatically cycles between three and ten dwellings overnight. Due to the need for cooperation and thorough patient education, PD is chosen less frequently than HD [1,2]. Blood purification in PD occurs through the peritoneal membrane, which lines the patient’s abdominal cavity. The functional properties of the peritoneum vary between individuals and depend primarily on its degree of vascularisation, which affects both solute transport and ultrafiltration efficiency. Several other factors, including the volume of dialysate, the frequency of exchanges, and the concentration of osmotically active components, also impact the effectiveness of PD. Significantly, the functional properties of the peritoneal membrane may deteriorate over time as a consequence of sustained exposure to hyperosmolar glucose-based dialysis fluids. One of the primary pathological processes underlying these changes is protein glycation, which results in structural alterations in the membrane and reduced transport efficiency. Therefore, it is essential to monitor peritoneal membrane function both before initiating PD and during treatment. Assessment of peritoneal transport capacity is commonly performed with the peritoneal equilibration test (PET) [3]. The standard PET involves the intraperitoneal infusion of a 2.27% glucose solution and the measurement of creatinine concentrations in plasma and dialysate at baseline and four hours post-infusion. The dialysate-to-plasma creatinine ratio (D/P) serves as a marker of solute diffusion across the membrane, while the decline in glucose concentration reflects the efficiency of convective transport. Based on PET results, patients are stratified into one of four transport categories—slow/low (L), fast/high (H), average slow/low (LA), or average fast/high (HA)—which are essential for optimising individual PD treatment.

CKD patients, including those requiring maintenance dialysis, may be at an increased risk of vitamin deficiencies mainly due to poor and/or restricted diets (and, in PD treatment, also resulting due to a feeling of stomach fullness caused by filling the abdominal cavity with peritoneal dialysis fluid), protein energy wasting, anorexia, and dialysis loss [4]. Literature data describing the amounts of vitamins lost during dialysis remain limited and mainly concern HD therapy. Water-soluble vitamins are especially susceptible to removal via dialysis due to their small molecular size. Vitamin B_1_ (thiamine) is one of the most essential vitamins for regulating basic metabolism [5]. Since most studies do not provide quantitative estimates of thiamine in CKD subjects, it is not possible to accurately determine the daily requirement for this group of patients. In a study by Hung et al., ten HD patients with unexplained encephalopathy were administered intravenous thiamine. All of them had thiamine deficiency, confirmed by a marked response to thiamine supplementation and/or low serum levels of this vitamin, amounting to 36 ± 6 nM, whereas typical levels in the healthy population exceed 50 nM [6,7]. Our previous study demonstrated that thiamine diphosphate is extensively lost during a single HD session, with losses ranging from a few per cent to nearly 100% in some cases [7]. In turn, vitamin B_6_ (pyridoxine) is involved in the synthesis of red blood cells and histamine, as well as in amino acid metabolism and gene expression [4]. Several studies have reported data on vitamin B_6_ washout during dialysis, revealing that 24–56% of patients experience significant losses in vitamin concentration, with amounts being higher in HD than in PD. Furthermore, the use of high-flux dialysis membranes was associated with a loss of over 50% pyridoxine and a significant reduction in serum B_6_ levels, even among patients receiving vitamin supplementation [8,9,10].

Determining the presence of vitamins B_1_ and B_6_ in various biological samples is challenging due to their low molecular weight, high polarity, and sensitivity to environmental factors such as light, temperature, and pH. Vitamin B_1_ is particularly unstable in neutral-to-alkaline environments, where it undergoes hydrolytic and oxidative degradation. Its decomposition rate increases markedly at temperatures above 40 °C, and exposure to oxygen and light accelerates this process further [11,12]. Conversely, it is relatively stable under acidic conditions (pH ~3–5), with over 90% of its initial concentration retained during extended storage [12]. Pyridoxal phosphate (PLP), the biologically active form of vitamin B6, is also prone to degradation under extreme pH or thermal stress and is particularly sensitive to light. Additionally, PLP binds tightly to proteins such as haemoglobin and albumin, limiting its availability in free form and complicating its detection in biological fluids [12,13]. These physicochemical features necessitate strict control of analytical conditions to ensure quantitative accuracy, particularly during sample collection, storage, and extraction. High-performance liquid chromatography (HPLC), coupled with various detection techniques, remains the most widely used approach for quantifying vitamers, offering generally high sensitivity, selectivity, and precision at a relatively low cost. Fluorescence detection is the most commonly used method for both B_1_ and B_6_ vitamers due to its high sensitivity, which is several orders of magnitude higher than that of common UV-Vis absorption and DAD detectors. Pre- or post-column derivatisation of thiamine into the fluorescent derivative thiochrome, typically using an alkaline potassium ferricyanide solution, yields detection limits (LODs) of 0.08 ng/mL, with quantification limits (LOQs) as low as 0.21 ng/mL for thiamine phosphates [14,15,16]. For B_6_ vitamers, native fluorescence or derivatisation with semicarbazide or sodium hydrosulfite improves sensitivity. Reported detection limits range from 1 to 27 ng/mL, and quantification limits range from 4 to 91 ng/mL, with linearity typically observed between 50 and 1800 ng/mL, depending on the vitamer type and the derivatisation method applied [17,18,19]. However, UV-Vis and DAD detectors, while less sensitive, offer robust and accessible alternatives with typical wavelength ranges of 245–280 nm for B_1_ vitamers and 254–290 nm for B_6_ vitamers. Sensitivity is 2–3 orders lower than fluorescence, but acceptable linearity and quantification in pharmaceutical formulations are still achievable [20,21,22]. On the other hand, electrochemical detectors, though less frequently used, provide good selectivity. Exemplary, coulometric detection has yielded LODs comparable to fluorimetry (e.g., 9.2 ng/mL for thiamine and 2.7 ng/mL for pyridoxine) with recovery rates above 99% [23,24]. Chemiluminescence detection, although rarely applied due to system complexity, offers excellent sensitivity down to sub-nanomolar levels. For example, a validated CL-HPLC method reports LODs of 0.61–4.76 nM for thiamine and its phosphate esters with recoveries of 96–103% [25]. Finally, mass spectrometry (MS), particularly when coupled with electrospray ionisation (e.g., ESI-QTOF) and operated in multiple reaction monitoring (MRM) mode, provides unmatched sensitivity and selectivity. MRM transitions enable the differentiation of vitamers with similar structures (e.g., thiamine esters) while maintaining low detection limits and a short analysis time. This technique is increasingly favoured in clinical diagnostics nowadays [26,27,28,29].

**Figure 1:**
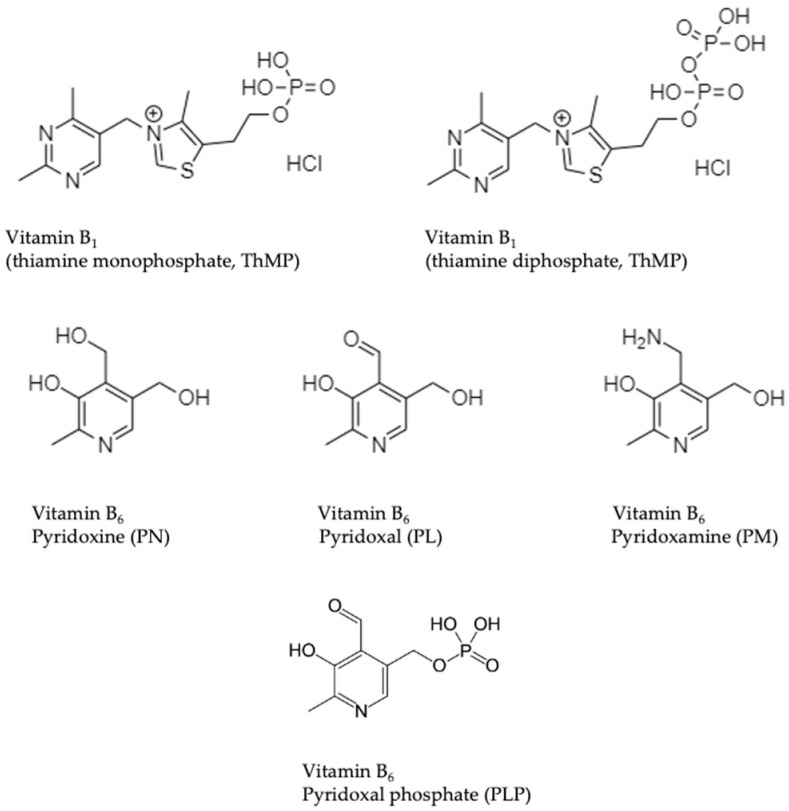
Chemical structures of the vitamers of vitamin B_1_ (thiamine monophosphate and thiamine diphosphate) and vitamin B_6_ (pyridoxal, pyridoxal phosphate, pyridoxamine, and pyridoxine) [12].

The main objective of this study was to evaluate the extent of loss of vitamins B_1_ and B_6_ during a single PD session in patients undergoing CAPD or APD treatment. Our focus was on quantifying thiamine monophosphate (ThMP), thiamine diphosphate (ThDP), pyridoxine (PN), pyridoxal (PL), and pyridoxamine (PM) in dialysis effluents. The chemical structures of the investigated B_1_ and B_6_ vitamers are presented in Figure 1. To this end, we developed and optimised a highly sensitive analytical method based on high-performance liquid chromatography with fluorescence detection (HPLC/FL), supplemented by LC-MS/MS for confirmation and specificity. A previous study by Jankowska et al. demonstrated the presence of ThDP in peritoneal effluent, confirming that biologically active thiamine can be eliminated during dialysis [30]. However, the overall extent of vitamin B loss and the contribution of other B_1_ and B_6_ vitamers remain insufficiently characterised, as do their clinical correlates. To address this, we employed analytical and chemometric methodologies, including multiple regression analysis (MRA), to identify clinical, biochemical, and demographic predictors of vitamin elimination. The aim of this study was to expand the understanding of micronutrient deficiency in PD patients and to provide a clinically relevant basis for optimising vitamin monitoring and supplementation in nephrology and internal medicine practice. This study is novel in its combined use of two complementary techniques—high-performance liquid chromatography–fluorescence detection (HPLC-FLD) and liquid chromatography–tandem mass spectrometry (LC-MS/MS)—to quantify multiple vitamers such as B_1_ and B_6_ in PD effluents simultaneously. To the best of our knowledge, this is one of the first reports to provide a quantitative assessment of these compounds side by side in a clinical CAPD/APD setting.

## 2. Results and Discussion

### 2.1. Participant Characteristics

A total of 41 participants were included in the study, and their general characteristics are shown in Table 1. Full clinical parameters characterising them are presented in Table S1. The patient group consisted of 21 females and 20 males, ranging in age from 20 to 88 years, with weights between 45.5 and 106.0 kg and heights between 150 and 195 cm. As mentioned previously, 22 patients were treated with CAPD and 19 with APD. According to the PET results, ten participants were classified as HA, 24 as LA, and seven as L transporters. The volume of dialysis fluid used for PD ranged from 1.650 to 15.800 mL. Some patients were anuric, while others had a 24 h urine output of less than 2.550 mL, indicating varying degrees of residual renal function. Dialysis adequacy, as assessed by Kt/V values, varied widely, ranging from 1.25 to 4.69. Estimated nutritional status, as determined by the nPCR, ranged from 0.67 to 2.25 g/kg/24 h.

After four hours of PD, BUN levels were low, ranging from 0.1 to 13.5 mg/dL, but these values exhibited significant variability after 24 h of dialysis, ranging between 3.04 and 219.40 mg/dL. Similarly, creatinine levels measured after 4 h of dialysis were lower than at the end of PD treatment, ranging from 0.10 to 2.20 mg/dL and from 0.80 to 37.80 mg/dL, respectively. Haemoglobin concentrations ranged from 7.3 to 14.0 g/dL, and haematocrit values varied between 23.4% and 42.5%. Peritoneal protein loss over a 24 h dialysis session ranged from 0.0 to 1.74 g, while leukocyte, lymphocyte, and neutrophil counts ranged from 2.84 to 12.43 × 10^9^/L, 0.44 to 2.95 × 10^9^/L, and 1.01 to 8.29 × 10^9^/L, respectively.

### 2.2. MS/MS Detection

Pseudo-molecular ions of target compounds could be detected in positive ESI ionisation mode. Declustering potential and collision energy were the most important parameters which influenced the mass spectrometer response. The most intense signal for ThDP was observed for one of the fragment ions of *m*/*z* 122. In contrast, for ThMP, the intensities for precursor and fragment ion *m*/*z* 122 were equal.

**Figure 2:**
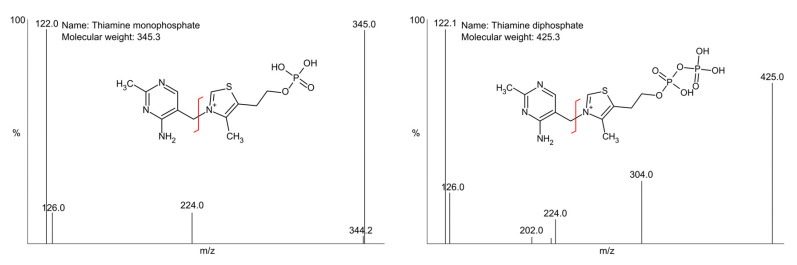
Mass spectra obtained in positive ionisation mode [M + H] ^+^ of the studied compounds and their fragmented structures.

For MS/MS, the FIA operating parameter was achieved using a 1 µg/mL solution of each substance. Mass spectra of the two analysed compounds obtained from the FIA mode are presented in Figure 2.

**Figure 3:**
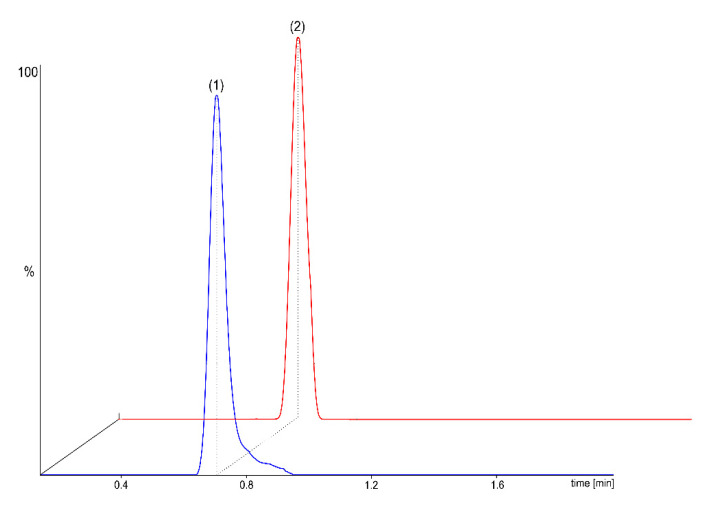
LC–MS/MS chromatogram of a mixture of two analytes in a standard sample, presenting chromatographic separation of ThMP (blue peak, marked as (1)) and ThDP (red peak, marked as (2)).

Analysed compounds are of different molecular masses and/or structures, so various transitions of parent ions to fragment ions were observed. However, co-elution was not avoided for ThMP and ThDP, whose structures differ only slightly (by one phosphate group). This can be neglected when applying MRM mode without a significant loss in method sensitivity. The proposed LC conditions (Table 5) enabled both qualitative and further quantitative analysis in biological samples, utilising MS/MS as the detection system. Applying the chromatographic conditions described above to a µLC column, the retention times for the three analytes, ThMP, ThDP, and PLP, were 0.71, 0.72, and 0.82 min, respectively. A chromatogram of a standard solution containing two targets is presented in Figure 3.

**Figure 4:**
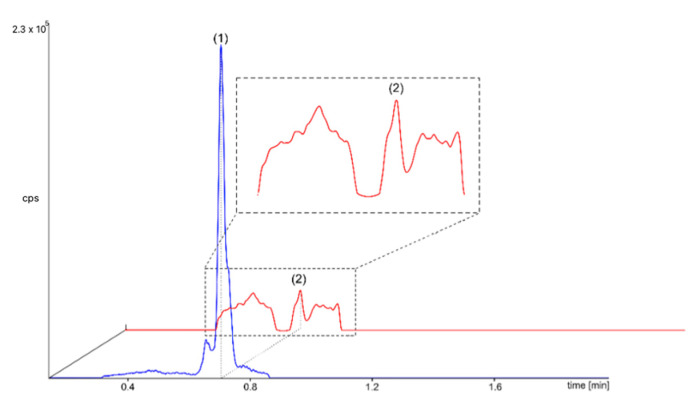
Chromatogram obtained for the native dialysis effluent sample containing ThMP (and ThDP signals detected (blue peak marked (1) and red peak marked (2), respectively).

To evaluate the presence of the investigated compounds in the peritoneal effluent samples, a few types of dialysates were randomly selected for analysis using the µLC-MS/MS technique. An example chromatogram of one of the samples, containing positive signals for the two target analytes, is presented in Figure 4. This experiment unequivocally demonstrates the application of this advanced technique to the analysis of native (i.e., significantly diluted) biomedical samples.

What is essential is that the results confirmed the presence of ThMP and ThDP signals in collected samples as early as the second hour of PET. These findings suggest that even a short medical procedure can cause the loss of vitamers from the patient’s body. However, PLP was not detected in dialysis effluent samples. The absence of detectable PLP in dialysate samples may be due to its intrinsic biochemical properties rather than to methodological limitations. PLP is known to bind strongly to plasma proteins, such as albumin and haemoglobin, which significantly reduces its presence in the free, dialyzable fraction [31]. In addition, its predominantly intracellular distribution and low free plasma concentrations may further restrict its passage across the peritoneal membrane.

Overall, the obtained results indicate that employing the LC-MS/MS technique with the described analytical parameters is a promising approach for detecting the B_1_ and B_6_ vitamers and could be successfully applied in modern analysis.

### 2.3. HPLC/FL Detection

#### 2.3.1. Vitamer Standards Analysis Using Proposed HPLC/FL Assays

**Figure 5:**
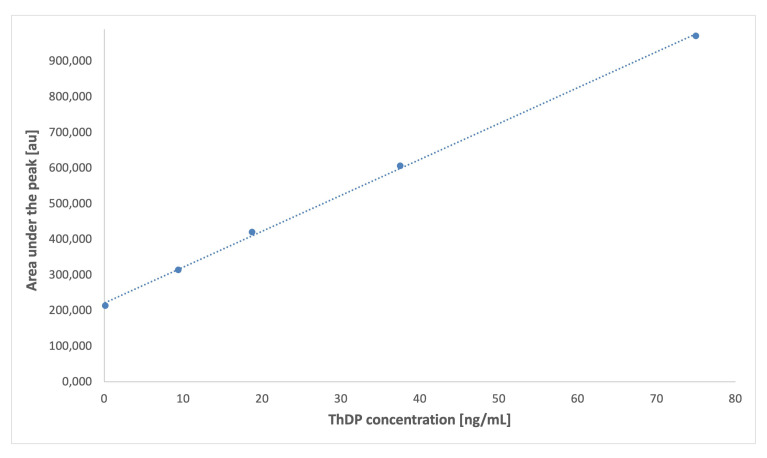
Representative calibration graph for thiamine diphosphate (ThDP) obtained using the HPLC/FL method.

Parameters characterising the proposed HPLC/FL assays for standards of ThMP, ThDP, PN, PL, and PM were obtained from calibration graphs and are presented in Table 2. Representative calibration graph for ThDP is presented in Figure 5. For ThMP and ThDP, the calibration graphs exhibit good linearity across a wide vitamer concentration range (0.12–75.0 ng/mL for ThMP and 0.15–75.0 ng/mL for ThDP), with Pearson’s correlation coefficients (R) of 0.9968 and 0.9983, respectively. Additionally, the obtained coefficients of variation of less than 5% confirm good precision of the method. In turn, the low LOD and LOQ values indicate the high sensitivity needed to detect and determine the low concentrations of vitamers in dialysis effluents. Similarly, the developed methods for PN, PL, and PM showed satisfactory parameters. Calibration graphs for these analytes were linear in the range of 1.50–50.0 ng/mL, with Pearson’s correlation coefficients ranging from 0.9928 (PN) to 0.9973 (PL). The precision, as expressed by CV values, was also acceptable, ranging from 1.76% (PM) to 3.94% (PL). The LODs for PN, PL, and PM were 0.03, 0.01, and 0.03 ng/mL, respectively, with corresponding LOQs of 0.09, 0.05, and 0.08 ng/mL. These results confirm that the method has sufficiently high sensitivity for determining B_6_ vitamers at trace amounts.

Since diluted standards were used for linearity, recovery studies were additionally performed. The results of these tests are presented in Table S2. For all analytes, recoveries were calculated based on the difference between the measured concentrations after spiking and the concentrations in the non-spiked samples. For ThMP, recoveries ranged from 91.4% to 97.9% across the concentration range of 10.0–50.0 ng/mL, with an average recovery of 94.7 ± 5.0%. ThDP showed similarly consistent performance, with recoveries ranging from 89.6% to 96.0%, and an average recovery of 93.1 ± 4.9%. PN also demonstrated good recovery, averaging 93.2 ± 4.2%, with individual recoveries ranging from 91.2% to 94.9%. In the case of PL, recovery values ranged from 89.6% to 93.9%, with an overall average of 92.3 ± 5.4%. PM showed slightly lower recovery at the lowest concentration tested (87.8%) but maintained acceptable precision across the tested range (10.0–25.0 ng/mL), with an average recovery of 92.1 ± 5.4%. Overall, the obtained recovery values for all analytes fall within the generally accepted range of 85–115% for bioanalytical methods, confirming the method’s reliability and applicability for quantifying vitamin B_1_ and B_6_ vitamers in dialysis effluent. The relatively low standard deviations (below 6% in all cases) indicate good reproducibility and precision of the method under the described experimental conditions.

#### 2.3.2. Dialysis Effluent Samples Analysis Using Proposed HPLC/FL Assays

Retention times for ThMP and ThDP ranged from 1.5 to 2.3 min and 2.8 to 3.5 min, respectively. For PN, PL, and PM, the ranges were 1.0 to 1.6 min, 2.0 to 2.6 min, and 3.8 to 4.5 min, respectively.

Based on the findings obtained for vitamin standards, the proposed HPLC/FL methods were applied to determine B_1_ and B_6_ vitamers in dialysis effluent samples. Summarised results of these analyses are presented in Table 3; the detailed data are shown in Tables S3 and S4.

The concentration values of the determined vitamins depend on the time at which the samples were collected. Levels of ThMP and ThDP were highest in probe A, reflecting the sample collected after a 24 h peritoneal dialysis session. In contrast, significantly lower levels were observed in samples B (the second hour of dialysis) and C (the fourth hour of dialysis), particularly for ThDP (0.45–31.16 ng/mL in probe B and 2.17–40.14 ng/mL in probe C versus 5.12–50.24 ng/mL in probe A). Similar time-dependent results were observed for B_6_ vitamers. The obtained PN, PL and PM concentrations in probe A (0.65–2.61 ng/mL; 5.83–17.26 ng/mL and 0.46–3.64 ng/mL, respectively) decreased in probe C (0.30–1.81 ng/mL; 1.54–16.20 ng/mL and 0.23–2.67 ng/mL, respectively) and successively in probe B (0.08–1.56 ng/mL; 0.09–16.63 ng/mL and 0.08–1.33 ng/mL, respectively).

**Figure 6:**
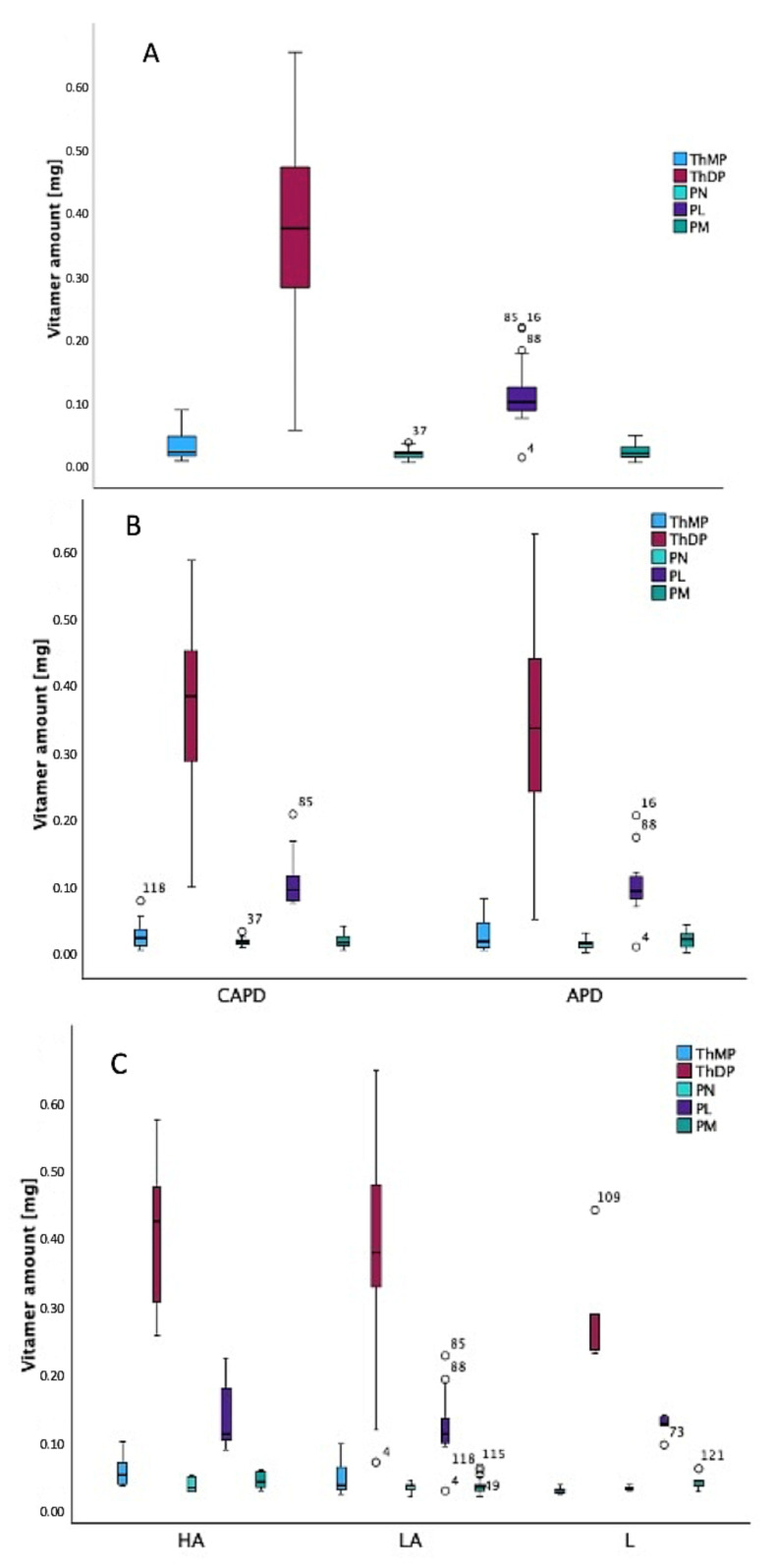
The amounts (in mg) of vitamers washed out during a 24 h dialysis session: overall, regardless of PD type (**A**), depending on PD type (**B**), and according to the category of peritoneal transport (**C**).

Knowing the volume of dialysis fluids used for 24 h dialysis (Table S1), the amounts of vitamins washed out were calculated and presented in Figure 6.

Figure 6A shows the amounts of vitamers lost for all patients, regardless of PD type. A quantitative analysis of the levels of vitamin B_1_ and B_6_ vitamers removed during a single 24 h dialysis session revealed wide interindividual variability. ThDP levels ranged from 0.048 mg to 0.573 mg, and ThMP levels varied from 0.005 mg to 0.076 mg. Among the B_6_ forms, PL had the highest removal concentration, ranging from 0.010 mg to 0.191 mg, whereas PN and PM were present in lower amounts, ranging from 0.003 to 0.030 mg and from 0.003 to 0.040 mg, respectively. This distribution is consistent with a fact described in the literature, indicating that PL (with PLP) is the most prevalent circulating form of vitamin B_6_ and is therefore more susceptible to dialysis-associated losses [32,33].

Figure 6B demonstrates loss of vitamers depending on the applied peritoneal dialysis type. In the CAPD group, ThMP amounts ranged from approximately 0.0050 to 0.0723 mg. In the APD group, meanwhile, values ranged from around 0.0050 to 0.0757 mg. ThDP, which is the biologically active coenzyme form of thiamine, exhibited greater variability across the two groups. CAPD patients had ThDP amounts ranging from 0.0724 to 0.5373 mg, while APD patients had amounts ranging from 0.0476 to 0.5727 mg. Although the maximum values were higher in the APD group, the overall distribution appeared more concentrated in CAPD patients. The three forms of vitamin B_6_, PN, PL, and PM, also exhibited noteworthy trends. PN losses were lower overall in the APD group, ranging from 0.0025 to 0.0283 mg; in CAPD, they ranged from 0.0085 to 0.0303 mg. PL, the metabolically active form of vitamin B_6_, showed a broader range in both groups; however, APD patients tended to have slightly lower minimum values again. The same trend was observed with PM, which plays a role in amino acid metabolism. CAPD patients had PM levels ranging from approximately 0.0048 to 0.0383 mg, whereas APD patients had levels ranging from 0.0026 to 0.0400 mg, suggesting greater variability in the latter group. While the mean values between CAPD and APD were not strikingly different for most vitamers, it is essential to highlight that the APD group often showed slightly more extreme minimum or maximum washed-out levels. Mann–Whitney U tests were performed for each vitamer concentration to assess whether differences between CAPD and APD were statistically significant [34]. Although trends were consistent with the visual interpretation of Figure 6B, no statistically significant differences were observed between the groups (*p* > 0.05).

Figure 6C presents the amounts of vitamin losses according to the peritoneal transport category revealed during the PET. For the HA group, ThMP values were available for six out of ten patients, ranging from approximately 0.0161 to 0.0757 mg. ThDP values were available for all ten HA patients, ranging from a minimum of approximately 0.1200 mg to a maximum of around 0.5078 mg. In turn, PN, PL, and PM ranged from 0.0076 to 0.0303 mg, 0.0595 to 0.1884 mg, and 0.0099 to 0.0383 mg, respectively. A greater number of observations were available in the LA group: 19 for ThMP and 24 for each of the other vitamins. ThMP ranged from 0.0050 to 0.0723 mg, while ThDP varied from 0.0476 to 0.5727 mg, reaching the highest amounts observed across all groups. PN values ranged from 0.0025 to 0.0235 mg, PL from 0.0096 to 0.1905 mg, and PM from 0.0026 to 0.0400 mg. These wide ranges, particularly for ThDP and PL, suggest that patients in the LA group experienced greater variability in vitamin loss. The L group had fewer observations overall (5−7 per compound). ThMP was determined to be between 0.0050 mg and 0.0174 mg. ThDP ranged from 0.1115 mg to 0.3860 mg, indicating more moderate levels compared to the HA and LA groups. Meanwhile, PN ranged from 0.0096 to 0.0190 mg, PL from 0.0710 to 0.1116 mg, and PM from 0.0084 to 0.0390 mg.

As mentioned earlier, PD patients are characterised as being at an elevated risk of water-soluble vitamin deficiencies, including B_1_ and B_6_. This is due to a combination of reduced dietary intake, chronic inflammation, and continuous losses via the peritoneal fluid. Our studies show that daily losses through the dialysate can be substantial, ranging from 0.053 to 0.649 mg/day for thiamine and from 0.016 to 0.261 mg/day for vitamin B_6_. These values are significant when compared to the physiological urinary excretion of healthy individuals, which is approximately 0.1–0.3 mg/day for both vitamins [35]. Therefore, PD patients may lose an equivalent or greater amount through dialysis than a healthy kidney would typically excrete. Furthermore, these losses represent a significant proportion of the Recommended Dietary Allowances (RDAs) of vitamins, 1.1–1.2 mg/day for thiamine and 1.3−1.7 mg/day for pyridoxine [36]. Accordingly, dialysis patients may lose 30–60% of the RDA of vitamins B_1_ and B_6_ through dialysate alone, without accounting for reduced intake or malabsorption. These findings are consistent with those of Boeschoten et al., who reported comparable or greater losses of vitamin B_6_ in PD patients than in healthy individuals and concluded that 2 mg/day of B_6_ supplementation was necessary to maintain normal plasma levels [37]. Furthermore, while evidence regarding vitamin B_1_ is more limited, studies have indicated that CKD patients, particularly those undergoing dialysis, may also be at risk of this vitamin. Clase et al. highlighted that reduced dietary intake, losses during dialysis, and altered distribution or metabolism may all contribute to insufficient thiamine status [38]. Similarly, Boeschoten et al. emphasised the need to monitor thiamine levels in CAPD patients and consider low-dose supplementation, particularly in those with poor nutritional status or symptoms suggestive of deficiency [37]. Dietary assessments by Martín-del-Campo et al. confirmed that only 45% of PD patients met their vitamin B_1_ requirements and that malnourished patients averaged just 0.83 mg of vitamin B_6_ per day, which is well below the RDA. These deficits were significantly associated with inflammation and poor nutritional status [39]. As mentioned earlier, Hung et al. reported that thiamine deficiency was the underlying cause of unexplained encephalopathy in ten out of 30 dialysis patients. Despite receiving oral B-complex supplementation, the patients remained deficient, suggesting that standard doses may be insufficient in cases of inflammation or altered absorption. Intravenous thiamine was required for neurological recovery, and delayed administration resulted in fatal outcomes in some cases [6]. Clinical guidelines support routine supplementation of water-soluble vitamins in dialysis patients, generally recommending: 1.1–1.5 mg/day of thiamine; 5–10 mg/day of vitamin B_6_, with higher doses warranted in cases of malnutrition, inflammation, or overt deficiency [32]. Our findings support these recommendations and also suggest a personalised supplementation protocol tailored to each patient.

### 2.4. Chemometric Analysis

The MRA was conducted to investigate the potential factors contributing to water-soluble vitamin losses during PD treatment. This approach enables the simultaneous evaluation of multiple independent variables, facilitating a comprehensive understanding of the complex interrelationships that affect vitamer concentrations in the dialysate. The dependent variables in this analysis were the concentrations of ThMP, ThDP, PN, PL, and PM vitamers in the dialysis effluents. The independent variables included a range of demographic, clinical, and biochemical factors (Table S1). Demographic variables comprised age, sex, weight, and height of the participants. Clinical variables included dialysis type, residual renal function (estimated by creatinine clearance), volume of dialysis fluid used for single dialysis peritoneum transport characteristics, and biochemical parameters, including haemoglobin (Hb), haematocrit (Hct), white blood cell level, lymphocytes, and neutrophil levels. A separate MRA model was constructed for each vitamer separately, based on the results obtained for samples collected after a 24 h dialysis session (Tables S3 and S4). Variables with a *p*-value of less than 0.05 in the univariate analysis were then entered into the multivariate model. Model performance was evaluated using the coefficient of determination (R^2^), residual diagnostics, and multicollinearity assessments through variance inflation factor (VIF) analysis. Statistically significant correlations were identified only for ThDP; no statistically reliable models were established for other vitamers. The results of multiple linear regression for ThDP are summarised in Table 4.

The final model included four variables: body weight, Hb, Hct, and creatinine concentration after 4 h (KD0). The model was statistically significant (F (4, 19) = 6.561, *p* = 0.002), explaining 58.0% of the variance in ThDP levels (R^2^ *=* 0.580, adjusted R^2^ *=* 0.492), with a standard error of estimate of 0.101. An R^2^ of 0.58 was interpreted as indicating a moderate to strong model fit, given the biological variability of the patient population [40]. All assumptions for multiple regression were satisfied, supporting the robustness of the ThDP model.

Among the predictors, body weight demonstrated the strongest positive association with ThDP (β = 0.771, *p* < 0.001), indicating that individuals with higher body mass may exhibit greater absolute ThDP levels. Additionally, Hb (β = 0.275, *p* = 0.041) was positively associated, and Hb showed a significant relationship (β = 0.255, *p* = 0.023). KD0 failed to prove a statistically substantial predictor (β = 0.284, *p* = 0.091); however, a moderate effect size was exhibited by this parameter. The finding that body weight significantly predicts ThDP levels could be associated with the distribution of thiamine in the human body. Because approximately 50–60% of the total vitamin B_1_ is stored in muscles, the differences in muscle mass could presumably explain this fact [41]. On the other hand, nearly 90% of circulating thiamine (mainly as ThDP) is contained in erythrocytes; thus, in the obtained model, haemoglobin and haematocrit levels correlate with thiamine diphosphate loss during dialysis session [7].

## 3. Materials and Methods

### 3.1. Reagents and Standard Solutions

ThMP, ThDP, PL, PM, pyridoxal 5′-phosphate (PLP), pyridoxine hydrochloride (PN), dithiothreitol, and methyl *tert*-butyl ether were of analytical grade and purchased from Sigma-Aldrich (Darmstadt, Germany). Methanol and acetonitrile, HPLC grade, were acquired from Carl Roth (Karlsruhe, Germany). Formic acid was purchased from Honeywell (Charlotte, NC, USA). Phosphoric acid, potassium dihydrogen phosphate, sodium dihydrogen phosphate, hydrochloric acid, and trichloroacetic acid (TCA) were acquired by Polish Chemical Reagents (Gliwice, Poland). Ultrapure water was provided by a Milli_Q purification system (Millipore, Burlington, MA, USA).

Stock solutions of ThMP and ThDP (150 ng/mL) were obtained by dissolving each vitamer in a mixture of 25 mM NaH_2_PO_4_ and methanol (90/10, *v*/*v*). Stock solutions containing individual B_6_ vitamers, PL, PN, PM (150 ng/mL) were prepared in a mixture of 150 mM KH_2_PO_4_ and acetonitrile (95:5, *v*/*v*). All stock solutions were stored at 4 °C in the dark before chromatographic runs.

### 3.2. Ethical Statements

The study was approved by the Bioethical Committee for Scientific Research at the Medical University of Gdańsk (No. NKBBN/217/2014). It was conducted by the World Medical Association’s 1964 Declaration of Helsinki and the EU rules of Good Clinical Practice. Informed consent was obtained from all participants involved in the research [42].

### 3.3. Participant Characteristics

Forty-one adult participants were recruited for the investigation. Among them, 22 individuals were treated with CAPD and 19 with APD. All patients underwent blood laboratory tests, including complete blood count (CBC) and selected biochemical parameters to assess their clinical conditions. CBCs were performed using flow cytometry (Sysmex XE 2100D, Sysmex Corp., Kobe, Japan). At the same time, creatinine and blood urea nitrogen (BUN) levels were determined using an automated analyser (Modular Roche, Roche Diagnostics, Indianapolis, IN, USA) via spectrophotometry for creatinine and a colourimetric kinetic assay for BUN, respectively. Creatinine levels, in addition to those assessed in PET (at baseline and four hours), were also measured 24 h after the PD treatment. BUN levels were determined at the beginning, after four hours, and at the end of dialysis to evaluate dialysis efficiency and adequacy (Kt/V). Additionally, patient medical characteristics included assessment of normalised protein catabolism ratio (nPCR) and calculation of glomerular filtration rate (GFR). Demographic parameters, including sex, age, weight, height, and dialysis duration, were also included in the analyses.

Total vitamin loss was measured in dialysis effluent samples after 24 h of the PD treatment (Samples A). To determine the dynamics of vitamin loss, samples were also examined at the second (Samples B) and fourth (Samples C) hour of dialysis.

### 3.4. Sample Preparation

Dialysis effluent samples were collected in light-protected vials (Eppendorf, Hamburg, Germany) and stored at −40 °C. They were gradually thawed, one at a time, immediately before chemical treatment and injection. To prepare the sample for chromatographic runs, 200 µL of dialysis effluent was transferred to a light-protected vial, and 400 µL of 0.8 M TCA was added. After vortexing (15 s), the vial was left standing on ice for 15 min. The precipitated samples were centrifuged at 13,000× *g* for 6 min at 10 °C. Finally, they were washed with water-saturated methyl *tert*-butyl ether (500 µL). Such extracts were used for the detection of studied vitamers, PLP and PL by µLC-MS/MS, and PL, PN, and PM by HPLC/FL. For the analysis of ThMP and ThDP by HPLC/FL, the extracts were derivatised according to the procedure described in our previous study [7]. Specifically, 80 µL of freshly prepared potassium ferricyanide (2 × 10^−3^ M) in 15% sodium hydroxide and 100 µL of the sample supernatant were added to a new light-protected vial. Next, 20 µL of methanol (for HPLC) and 100 µL of 0.01 M dithiothreitol (DTT) solution were added to each vial. After vortexing, the mixtures were set aside for 15 min before being subjected to HPLC runs.

### 3.5. Micro LC-MS/MS Conditions

The µLC analyses were performed employing an Eksigent microLC200 system (part of AB Sciex, Darmstadt, Germany). The analytes were separated on a HALO C18 column (50 mm × 0.5 mm, 2.7 µm; Advanced Materials Technology, Wilmington, DE, USA). The mobile phase A (ultrapure water) and B (acetonitrile) contained 0.1% (*v*/*v*) formic acid at a flow rate of 30 µL/min. The gradient started from 95% A to 5% A in 2.5 min, then returned to 95% A in 0.5 min, and equilibrated at 95% A for 15 min. The column was maintained at 30 °C, and the sample volume injected was 2 µL.

The µLC system was coupled directly to a QTRAP 6500 triple quadrupole MS (AB Sciex, Darmstadt, Germany), equipped with an electrospray (ESI) interface, operated in positive ionisation mode. Compound dependent MS parameters (declustering potential (DP), collision energy (CE), collision cell exit potential (CXP) and entrance potential (EP)) as well as compound multiple reaction monitoring (MRM) transitions were optimised by direct infusion of individual standard solutions at 1 µg/mL. All transitions were recorded using the scheduled MRM algorithm with a 30 s detection window. Source-dependent parameters, including curtain gas (CUR), source temperature (TEM), ion spray voltage (IS), nebuliser gas (GS1), and turbo gas (GS2), were determined using Flow Injection Analysis (FIA). Conditions for the monitored ion transitions and MS/MS operation parameters are presented in Table 5. All data were collected and processed using the analyst software.

The LC-MS/MS method was not fully validated due to the qualitative nature of its application in this study. It was used to confirm the identity of the B_1_ and B_6_ vitamer in selected peritoneal dialysis effluent samples, to support the primary quantitative analysis conducted using HPLC with fluorescence detection. Key qualitative performance parameters, including MRM transition specificity, retention time stability and ion ratio consistency (Q/q), were carefully monitored using reference standards. These controls ensured reliable compound identification, despite the absence of full quantitative validation.

### 3.6. HPLC/FL Conditions

B_1_ and B_6_ vitamers analysis in dialysis effluents was performed in triplicate for all samples (A, B and C) employing an isocratic HPLC system (Waters Corporation, Milford, MA, USA) with a W600 two-channel pump, a controller with a degassing unit (He), a 7725i Rheodyne injection loop, and a W474 fluorescence detector. A Gemini 5u C6-Phenyl reverse-phase column (150 × 4.6 mm, 5 µm, Phenomenex) was used for all HPLC runs. Waters Empower 2 software (Waters Corporation, Milford, MA, USA) was applied to collect and analyse the chromatographic data.

Chromatographic analyses of B_1_ vitamers were performed at a flow rate of 1.0 mL/min (ambient temperature), with excitation and emission wavelengths of the fluorescence detector set at 375 nm and 440 nm, respectively, and moderate sensitivity (100 mV). The injection volume was set to 50 µL for each run. The mobile phase consisted of a mixture of dibasic sodium phosphate (25 mM) and methanol (9/1, *v*/*v*) with 85% phosphoric acid adjusted to pH 3.5.

B_6_ vitamers chromatographic analyses were carried out at a flow rate of 1.2 mL/min. (ambient temperature), an excitation/emission wavelength of the fluorescence detector of 295 nm/405 nm, and moderate sensitivity (100 mV). The injection volume was set to 50 µL for each run. The mobile phase consisted of a mixture of dibasic potassium phosphate (150 mM), sodium bisulfide (5 mM), and acetonitrile in a 50/45/5 (*v*/*v*/*v*) ratio.

### 3.7. HPLC/FL Method Validation

To validate the method, linearity, recovery, accuracy, LOD, and LOQ were assessed [43]. Linearity was evaluated by constructing calibration graphs for each standard vitamer solution. Five-point graphs were prepared by plotting the chromatogram area values under the peaks for different vitamer concentrations. The Pearson correlation coefficient was calculated as the covariance between the two variables divided by the product of their standard deviations [7]. Recovery and accuracy were investigated by spiking effluent samples with standard vitamer solutions. The percentage recovery was calculated by comparing the measured concentrations with the known added concentrations of the target analytes (vitamers), thereby expressing accuracy [7]. The LOD values were calculated based on the standard deviation of the response (S_y_) and the slope (S) of the calibration curve using the following formula:

$$\mathrm{LOD}\;=\;3.3\;\times \;(\frac{S_{y}}{S})$$

The LOQ values were determined similarly, using 10 times the standard deviation of the response:

$$\mathrm{LOQ}\;=\;10\;\times \;(\frac{S_{y}}{S})$$

### 3.8. Statistical Analysis

The MRA was applied as a chemometric tool to investigate potential demographic, clinical, and biochemical parameters that may be significant for assessing vitamers washout during a single peritoneal dialysis session [44]. The validity of each regression model was confirmed. Homoscedasticity (i.e., constant variance of residuals across levels of the independent variables) of the data was evaluated by plotting standardised residuals versus fitted values and tested using the Breusch–Pagan test [45]. The normality of the residuals was assessed using Q–Q plots and the Shapiro–Wilk test [46]. Multicollinearity among the predictors was examined using the variance inflation factor (VIF); VIF values above five were considered to indicate concerning levels of collinearity [47,48]. Model fit was evaluated based on adjusted R^2^ values. A *p*-value of less than 0.05 was considered statistically significant. All statistical tests were two-tailed. All statistical analyses were performed using IBM SPSS Statistics (version 30.0.0.0).

## 4. Limitations

This study was limited to assessing the loss of vitamins B_1_ and B_6_ via peritoneal dialysate during a single dialysis session. As the study was designed to quantify dialysate-based elimination specifically, plasma vitamin levels were not analysed. While this approach enables the estimation of typical daily losses, it does not consider cumulative depletion over longer periods. It would be valuable for future studies to incorporate repeated measurements across multiple sessions or 24–hour dialysate collections to assess whether sustained losses significantly impact the long-term vitamin balance in dialysis patients.

## 5. Conclusions

This study demonstrates that significant quantities of the water-soluble vitamins B_1_ and B_6_ (including their biologically active forms, notably ThDP and PL) are lost during a single peritoneal dialysis session. The HPLC/FL method developed for this study exhibited excellent analytical performance, demonstrating low coefficients of variation, low detection limits, and broad linearity ranges. Independent confirmation using µLC-MS/MS supported the specificity and reliability of the findings. Based on chemometric modelling, body weight, haemoglobin concentration, and haematocrit were identified as significant predictors of ThDP elimination.

These findings support the usefulness of the proposed HPLC/FL assay for monitoring vitamin losses in clinical and research settings. From an internal medicine perspective, the results highlight a potentially under-recognised contributor to micronutrient deficiency in dialysis patients, emphasising the need to reassess current empirical approaches to vitamin supplementation in peritoneal dialysis.

## Figures and Tables

**Table 1:** Basic clinical characteristics of participants under study.

Characteristic (Parameter)	Range
Age [years]	20–88
Weight [kg]	45.5–106.0
Height [cm]	150–195
Volume of dialysis fluid [mL]	1650–15.800
Urine output [mL/24 h]	0–2550
Kt/V	1.25–4.69
nPCR [g/kg/24 h]	0.67–2.25
BUN—after 4 h [mg/dL]	0.1–13.5
BUN—after 24 h [mg/dL]	3.04–219.40
Creatinine—after 4 h [mg/dL]	0.10–2.20
Creatinine—after 24 h [mg/dL]	0.80–37.80
Haemoglobin [g/L]	7.3–14.0
Haematocrit [%]	23.4–42.5
Protein peritoneal loss [g/ 24 h]	0.0–1.74
White blood cells [×10^9^/L]	2.84–12.43
Lymphocytes [×10^9^/L]	0.44–2.95
Neutrocytes [×10^9^/L]	1.01–8.29

**Table 2:** The parameters characterising the proposed HPLC/FL assays for standards of ThMP, ThDP, PN, PL, and PM were obtained based on the calibration graphs.

ThMP		ThDP	PN	PL	PM
Tested Parameter	Value	Value	Value	Value	Value
Equation of the calibration graph (c—vitamer’s concentration, [ng/mL]	99,856 c + 5650	96,250 c + 6096	8350 c + 106	6971 c + 196	7338 c + 217
Linearity range [ng/mL]	0.12–75.0	0.15–75.0	1.50–50.0	1.50–50.0	1.50–50.0
Correlation coefficient (R)	0.9968	0.9983	0.9928	0.9973	0.9951
Coefficient of variation (CV), [%]	3.12	4.16	2.68	3.94	1.76
LOD [ng/mL]	0.08	0.02	0.03	0.01	0.03
LOQ [ng/mL]	0.21	0.07	0.09	0.05	0.08

**Table 3:** Summarised results of vitamers concentrations in dialysis effluent samples.

	Range [ng/mL]				
Probe	ThMP	ThDP	PN	PL	PM
A	0.53–6.88	5.12–50.24	0.65–2.61	5.83–17.26	0.46–3.64
B	0.53–1.66	0.45–31.16	0.08–1.56	0.09–16.63	0.08–1.33
C	0.43–3.72	2.17–40.14	0.30–1.81	1.54–16.20	0.23–2.67

**Table 4:** The summary of multiple regression for ThDP loss during single dialysis.

Predictor	B	SE B	β (Beta)	t	*p*-Value	95% CI for B
(Constant)	−0.121	0.242	−	−0.500	0.623	[−0.628, 0.386]
Weight	0.007	0.002	0.771	4.413	<0.001	[0.004, 0.010]
Haemoglobin (Hb)	0.255	0.103	0.255	2.472	0.023	[0.039, 0.470]
Hematocrit (Hct)	0.078	0.036	0.275	2.189	0.041	[0.003, 0.152]
KD0	0.087	0.049	0.284	1.778	0.091	[−0.015, 0.189]
Model Statistics: R^2^ = 0.580, Adjusted R^2^ = 0.492 F (4, 19) = 6.561, p = 0.002 Standard Error of Estimate = 0.101						

Abbreviations: B—Unstandardised regression coefficient; SE B—standard error of the unstandardised coefficient; β (Beta)—standardised regression coefficient; t—t-statistic for the significance of each predictor; *p*-value—probability value; indicates the level of statistical significance; 95% CI for B—95% confidence interval for B.

**Table 5:** Optimal conditions for the monitored ion transitions and MS/MS operation parameters.

Ion Transition Monitored Parameters								
Compound	Quantitative [Q] qualitative [q] parent ion > fragment ion *		DP (V)	EP (V)	CXP (V)		CE (V)	MRM ratio
PLP	Q 248 > 150 q 248 > 134		46	10	16 8		19 25	1.4
PL	Q 170 > 152 q 170 > 134		46	10	16 8		19 25	1.4
ThMP	Q 345 > 122 q 345 > 224		41	10	14 10		23 23	4.8
ThDP	Q 425 > 122 q 425 > 304		31	10	22 22		27 23	2.1
MS/MS Operation Parameters								
Compound	Quantitative [Q] qualitative [q] parent ion > fragment ion *	CUR (psi)		IS (V)	T (°C)	GS1 (psi)		GS2 (psi)
PLP	Q 248 > 150 q 248 > 134	30		5500	200	20		20
PL	Q 170 > 152 q 170 > 134	30		5500	200	20		20
ThMP	Q 345 > 122 q 345 > 224	20		5500	200	20		20
ThDP	Q 425 > 122 q 425 > 304	20		5500	300	30		20
Chosen parameters		20		5500	200	20		20

* Quantitative (Q) transitions refer to the most abundant and stable fragment ions used for compound quantification. Qualitative (q) transitions serve as confirmatory ions to ensure compound specificity. The consistency between Q and q signals, including their retention times and MRM ratios, supports the accurate identification of each vitamin vitamer.

## Data Availability

All relevant data can be found within the manuscript and Supplementary File.

## Supplementary Materials

The following supporting information can be downloaded at https://www.mdpi.com/article/10.3390/ijms26157177/s1.

## Abbreviations

The following abbreviations are used in this manuscript:

APD	Automated peritoneal dialysis
CAPD	Continuous ambulatory peritoneal dialysis
HPLC/FL	High-performance liquid chromatography with fluorescence detection
LOD	Limit of detection
LOQ	Limit of quantification
MRA	Multiple regression analysis
PET	Peritoneal equilibration test
PD	Peritoneal dialysis
PL	Pyridoxal
PLP	Pyridoxal 5-phosphate
PN	Pyridoxine
PM	Pyridoxamine
ThMP	Thiamine monophosphate
ThDP	Thiamine diphosphate
