# Supplementary material for: A Simultaneous Determination of the B1 and B6 Vitamers Reveals Their Loss During a Single Peritoneal Dialysis Session: Chromatographic and Chemometric Approach
Source: Int J Mol Sci. 2025 Jul 25;26(15):7177. doi: 10.3390/ijms26157177 (PMC12346733; doi:10.3390/ijms26157177)
Supplement: Supplementary file 1 [file ijms-26-07177-s001.zip › ijms-3764652-supplementary.pdf]

International Journal of
*Molecular Sciences*

MDPI

*Supplementary Materials*

# A Simultaneous Determination of the $B_1$ and $B_6$ Vitamers Reveals Their Loss During a Single Peritoneal Dialysis Session: Chromatographic and Chemometric Approach

**Paweł Rudnicki-Velasquez [1,*], Karol Krzymiński [2], Magdalena Jankowska [3], Anna Baraniak [4] and Paulina Czaplewska [5]**

[1] Department of Falsified Medicines and Medical Devices, National Medicines Institute, 00-725 Warsaw, Poland
[2] Faculty of Chemistry, University of Gdańsk, 80-308 Gdańsk, Poland; karol.krzyminski@ug.edu.pl
[3] Department of Nephrology, Transplantology and Internal Medicine, Medical University of Gdańsk, 80-210 Gdańsk, Poland; magdalena.jankowska@gumed.edu.pl
[4] Department of Pharmaceutical Microbiology and Laboratory Diagnostics, National Medicines Institute, 00-725 Warsaw, Poland; a.baraniak@nil.gov.pl
[5] Laboratory of Mass Spectrometry-Core Facility Laboratories, Intercollegiate Faculty of Biotechnology of UG and MUG, University of Gdańsk, 80-307 Gdańsk, Poland; paulina.czaplewska@ug.edu.pl
* Correspondence: p.rudnicki@nil.gov.pl; Tel.: +48-228-412-121

**Contents**

**Table S1.** Clinical data included in statistical analysis.

**Table S2.** Recoveries of ThMP, ThDP, PN, PL, and PM added to dialysis effluents.

**Table S3.** Levels of the thiamine monophosphate (ThMP, in ng/mL) and thiamine diphosphate (ThDP, in ng/mL) in dialysis fluids assessed using the proposed RP-HPLC/FL assay.

**Table S4.** Levels of the pyridoxine (PN, in ng/mL), pyridoxal (PL, in ng/mL) and pyridoxamine (PM, in ng/mL) in dialysis fluids assessed using the proposed RP-HPLC/FL assay.

**Table S1.** Clinical data included in statistical analysis.

| Pa-tient | Probe | Sex | Age | Weight | Height | DO type | Vd | Vm | Pro-tein D | BUN D | BUN D0 | K D | K D0 | Kt/V | nPCR | Hb | Hct | WBC | Ntf | Lmf |
|---|---|---|---|---|---|---|---|---|---|---|---|---|---|---|---|---|---|---|---|---|
| | A | | | | | | 1040 | | | | | | 0.3 | | | | 32. | | 4.6 | 1.6 |
| | | | | | | | 0 | 0 | 0.95 | 50.2 | 2 | 8.51 | 8 | 2.43 | 1.28 | 9.3 | 7 | 7.41 | 9 | 5 |
| P1 | B | | | | | CAP | | | | | | | | | | | | | | |
| | | F | 59 | 56 | 152 | D | | | | 23.3 | | 4.22 | | | | | | | | |
| | C | | | | | | | | | 37.7 | | 7.69 | | | | | | | | |
| | A | | | | | | | | | | | | 0.1 | | | | 34. | | 6.0 | 1.7 |
| | | | | | | | 1650 | 0 | 0.29 | 23.6 | 2 | 3.17 | 4 | 1.72 | 1.72 | 9.9 | 8 | 9.14 | 9 | 2 |
| P2 | B | F | 78 | 69 | 164 | APD | | | | 20.1 | | 3.04 | | | | | | | | |
| | C | | | | | | | | | 3.04 | | 6.49 | | | | | | | | |
| | A | | | | | | 1120 | | | | | | 0.9 | | | | 31. | 11.5 | 8.0 | 2.5 |
| | | | | | | | 0 | 0 | 0.47 | 60.1 | 6.7 | 8.08 | 8 | 1.98 | 1.23 | 9.9 | 9 | 4 | 2 | 1 |
| P3 | B | | | | | CAP | | | | | | | | | | | | | | |
| | | F | 27 | 66 | 164 | D | | | | 31.8 | | 4.64 | | | | | | | | |
| | C | | | | | | | | | 55.7 | | 8.17 | | | | | | | | |
| | A | | | | | | | 230 | | | | | | | | 12. | 41. | | 1.0 | 0.4 |
| | | | | | | | 6300 | 0 | 0.49 | 23.5 | 2 | 3.7 | 0.2 | 3.51 | 0.94 | 3 | 6 | 3.86 | 1 | 4 |
| P4 | B | | | | | CAP | | | | | | | | | | | | | | |
| | | F | 22 | 55.5 | 160 | D | | | | 11 | | 11 | | | | | | | | |
| | C | | | | | | | | | 22.2 | | 22 | | | | | | | | |
| | A | | | | | | | | | | | | 0.2 | | | 10. | 38. | | 5.1 | 1.3 |
| | | | | | | | 9500 | 800 | 0.64 | 45.5 | 2 | 6.65 | 6 | 2.02 | 0.88 | 3 | 9 | 7.48 | 9 | 4 |
| P5 | B | | | | | CAP | | | | | | | | | | | | | | |
| | | M | 61 | 77.4 | 164 | D | | | | 28.2 | | 3.9 | | | | | | | | |
| | C | | | | | | | | | 3.9 | | 6.1 | | | | | | | | |
| | A | | | | | | 1100 | | | | | | | | | | 29. | 12.4 | 8.0 | 2.9 |
| | | | | | | | 0 | 700 | 1.28 | 52.3 | 2.2 | 2.68 | 2.2 | 3.77 | 1.84 | 9.9 | 0 | 3 | 1 | 4 |
| P6 | B | F | 75 | 45.5 | 150 | APD | | | | 22.6 | | 1.27 | | | | | | | | |
| | C | | | | | | | | | 45.8 | | 3.07 | | | | | | | | |
| | A | | | | | | 1040 | | | | | | 0.9 | | | | 28. | | 6.6 | 1.4 |
| | | | | | | | 0 | 0 | 0.49 | 44.7 | 4.1 | 8.2 | 4 | 1.25 | 0.67 | 9.4 | 3 | 8.8 | 4 | 9 |
| P7 | B | M | 59 | 89 | 174 | APD | | | | 20.9 | | 5.14 | | | | | | | | |
| | C | | | | | | | | | 32 | | 8.68 | | | | | | | | |
| | A | | | | | | 1070 | | | | | | | | | 11. | 28. | | | |
| | | | | | | | 0 | 500 | 0.53 | 40.6 | 2 | 6.57 | 0.3 | 2.15 | 0.97 | 3 | 8 | 8.5 | 5.7 | 1.8 |
| P8 | B | | | | | CAP | | | | | | | | | | | | | | |
| | | F | 42 | 65.2 | 170 | D | | | | 26.4 | | 4.02 | | | | | | | | |
| | C | | | | | | | | | 42.6 | | 6.66 | | | | | | | | |
| | A | | | | | | 1110 | 110 | | | | | | | | 10. | 37. | | 5.6 | 2.3 |
| | | | | | | | 0 | 0 | 0.48 | 43.1 | 2 | 1.73 | 0.1 | 2.1 | 1.18 | 7 | 6 | 8.95 | 7 | 4 |
| P9 | B | F | 46 | 92.2 | 158 | APD | | | | 34.6 | | 1.29 | | | | | | | | |

| | | | | | | | | | | | | | | | | | | | | |
|---|---|---|---|---|---|---|---|---|---|---|---|---|---|---|---|---|---|---|---|---|
| | C | | | | | | | | | 54.1 | | 2.27 | | | | | | | | |
| | A | | | | | | | | | | | | | | | | 24. | | | 1.8 |
| | | | | | | | 9300 | 400 | 0.95 | 42.7 | 3.6 | 5.52 | 0.4 | 1.85 | 0.96 | 8 | 7 | 8.44 | 5.2 | 9 |
| P10 | B | | | | | CAP | | | | | | | | | | | | | | |
| | | M | 72 | 65.7 | 166 | D | | | | 28.8 | | 3.19 | | | | | | | | |
| | C | | | | | | | | | 44 | | 4.94 | | | | | | | | |
| | A | | | | | | 1140 | 100 | | | | | 0.3 | | | 11. | 40. | | 5.2 | 1.2 |
| | | | | | | | 0 | 0 | 1.15 | 49 | 6.7 | 2.66 | 3 | 1.95 | 1.29 | 9 | 3 | 7.56 | 8 | 1 |
| P11 | B | M | 57 | 92 | 176 | APD | | | | 48.2 | | 2.3 | | | | | | | | |
| | C | | | | | | | | | 71.4 | | 3.89 | | | | | | | | |
| | A | | | | | | | 200 | | | | | 0.5 | | | 10. | 33. | | 5.4 | 2.0 |
| | | | | | | | 8200 | 0 | 1.06 | 36.4 | 2.8 | 6.72 | 2 | 2.48 | 0.89 | 2 | 1 | 9.16 | 5 | 7 |
| P12 | B | | | | | CAP | | | | | | | | | | | | | | |
| | | M | 57 | 86.8 | 172 | D | | | | 22.6 | | 3.38 | | | | | | | | |
| | C | | | | | | | | | 33.5 | | 5.11 | | | | | | | | |
| | A | | | | | | 1160 | | | | | | 0.8 | | | 10. | 34. | | | 2.9 |
| | | | | | | | 0 | 500 | 0.91 | 51.2 | 6.4 | 8.79 | 6 | 1.75 | 1.02 | 4 | 8 | 5.09 | 1.2 | 5 |
| P13 | B | | | | | CAP | | | | | | | | | | | | | | |
| | | M | 29 | 67.5 | 185 | D | | | | 45.8 | | 5.26 | | | | | | | | |
| | C | | | | | | | | | | | 11.3 | | | | | | | | |
| | | | | | | | | | | 74 | | 5 | | | | | | | | |
| | A | | | | | | 1080 | | | | | | 0.3 | | | | 25. | | 2.6 | 1.4 |
| | | | | | | | 0 | 500 | 0.45 | 36.3 | 4.2 | 2.67 | 7 | 1.74 | 1.03 | 8.5 | 4 | 4.84 | 6 | 7 |
| P14 | B | F | 20 | 60 | 170 | APD | | | | 34.8 | | 3 | | | | | | | | |
| | C | | | | | | | | | 52.6 | | 5.04 | | | | | | | | |
| | A | | | | | | 1260 | | | | | | 0.3 | | | 11. | 39. | | 2.6 | |
| | | | | | | | 0 | 700 | 0.38 | 61.2 | 4 | 5.82 | 5 | 1.66 | 1.94 | 3 | 9 | 4.44 | 6 | 1.1 |
| P15 | B | M | 48 | 96 | 195 | APD | | | | 34.7 | | 3.52 | | | | | | | | |
| | C | | | | | | | | | 66 | | 7.22 | | | | | | | | |
| | A | | | | | | | 150 | 0.05 | | | | | | | | 28. | | | |
| | | | | | | | 7785 | 0 | 1 | 18.8 | 2 | 2.5 | 0.1 | 2.58 | 0.92 | – | 2 | – | – | – |
| P16 | B | F | 73 | 58 | 160 | APD | | | | 19.8 | | 2.47 | | | | | | | | |
| | C | | | | | | | | | 30.4 | | 4.08 | | | | | | | | |
| | A | | | | | | 1065 | 210 | 0.08 | | | | 0.2 | | | | 30. | | | |
| | | | | | | | 0 | 0 | 7 | 52 | 4.6 | 3.01 | 3 | 1.94 | 1.37 | 9.9 | 7 | 6.33 | 2.9 | 2.2 |
| P17 | B | M | 67 | 106 | 180 | APD | | | | 37.5 | | 2 | | | | | | | | |
| | C | | | | | | | | | 80.2 | | 4.4 | | | | | | | | |
| | A | | | | | | | 170 | | | | | 1.0 | | | 10. | 37. | | | |
| | | | | | | | 9200 | 0 | 1.74 | 72 | 8.8 | 9.55 | 9 | 2.14 | 1.29 | 9 | 6 | 6.14 | 5 | 0.6 |
| P18 | B | | | | | CAP | | | | | | | | | | | | | | |
| | | M | 33 | 75.5 | 185 | D | | | | 35.1 | | 4.04 | | | | | | | | |
| | C | | | | | | | | | 58.5 | | 7.26 | | | | | | | | |
| | A | | | | | | | 255 | 0.05 | | | | 0.1 | | | | 42. | | 5.7 | 1.6 |
| | | | | | | | 8580 | 0 | 6 | 37.1 | 2 | 3.6 | 2 | 3.6 | 1.03 | 14 | 5 | 8.23 | 4 | 6 |

| | | | | | | | | | | | | | | | | | | | | |
|---|---|---|---|---|---|---|---|---|---|---|---|---|---|---|---|---|---|---|---|---|
| P19 | B | | | | | CAP | | | | | | | | | | | | | | |
| | | M | 49 | 77 | 178 | D | | | | 18.8 | | 1.73 | | | | | | | | |
| | C | | | | | | | | | 31.4 | | 3.01 | | | | | | | | |
| | A | | | | | | | 150 | | | | | 0.1 | | | 11. | 37. | | | 2.3 |
| | | | | | | | 7100 | 0 | 0 | 50.4 | 0.1 | 4.65 | 7 | 2.2 | 2.25 | 8 | 3 | 8.28 | 4.9 | 7 |
| P20 | B | | | | | CAP | | | | | | | | | | | | | | |
| | | F | 40 | 45.7 | 158 | D | | | | 16.4 | | 1.56 | | | | | | | | |
| | C | | | | | | | | | 32.9 | | 3.41 | | | | | | | | |
| | A | | | | | | | 150 | 0.06 | | | | | | | 11. | 34. | | 5.6 | 1.9 |
| | | | | | | | 9200 | 0 | 5 | 48.1 | 2 | 3.55 | 0.1 | 2.86 | 1.33 | 1 | 7 | 9.31 | 3 | 1 |
| P21 | B | | | | | CAP | | | | | | | | | | | | | | |
| | | M | 72 | 78.2 | 166 | D | | | | 29.5 | | 1.9 | | | | | | | | |
| | C | | | | | | | | | 20.1 | | 3.34 | | | | | | | | |
| | A | | | | | | 1070 | 175 | 0.07 | | | | 0.1 | | | 12. | 36. | | 4.1 | 1.7 |
| | | | | | | | 0 | 0 | 9 | 34.9 | 2 | 2.91 | 6 | 3.31 | 1.09 | 4 | 4 | 7.08 | 6 | 8 |
| P22 | B | M | 55 | 95 | 174 | APD | | | | 18.8 | | 1.64 | | | | | | | | |
| | C | | | | | | | | | 34.2 | | 3.32 | | | | | | | | |
| | A | | | | | | | 200 | 0.07 | | | | 0.2 | | | 11. | 34. | | 3.6 | 0.9 |
| | | | | | | | 9500 | 0 | 6 | 40.6 | 3.3 | 3.13 | 8 | 4.14 | 1.5 | 2 | 8 | 5.1 | 1 | 3 |
| P23 | B | | | | | CAP | | | | | | | | | | | | | | |
| | | F | 55 | 67 | 158 | D | | | | 25.3 | | 1.65 | | | | | | | | |
| | C | | | | | | | | | 41.3 | | 3.13 | | | | | | | | |
| | A | | | | | | 1045 | 140 | | 219. | | | 0.3 | | | 11. | 34. | | 2.6 | 1.3 |
| | | | | | | | 0 | 0 | 0.18 | 4 | 4.3 | 37.8 | 5 | 2.16 | 1.16 | 4 | 9 | 4.53 | 5 | 1 |
| P24 | B | | | | | CAP | | | | | | | | | | | | | | |
| | | F | 65 | 75.2 | 158 | D | | | | 26.5 | | 1.94 | | | | | | | | |
| | C | | | | | | | | | 47 | | 3.66 | | | | | | | | |
| | A | | | | | | | 170 | 0.07 | | | | 0.2 | | | 11. | 36. | | 4.2 | |
| | | | | | | | 8800 | 0 | 9 | 40.1 | 4 | 2.53 | 6 | 4.69 | 1.49 | 4 | 8 | 6.46 | 6 | 1.2 |
| P25 | B | | | | | CAP | | | | | | | | | | | | | | |
| | | F | 49 | 63.4 | 158 | D | | | | 22.6 | | 1.21 | | | | | | | | |
| | C | | | | | | | | | 31.6 | | 1.81 | | | | | | | | |
| | A | | | | | | 1165 | | | | | | 0.4 | | | | 33. | | 3.1 | 1.4 |
| | | | | | | | 0 | 0 | 0.96 | 67.3 | 3.5 | 8.76 | 7 | 1.86 | 1.21 | 11 | 7 | 5.6 | 4 | 2 |
| P26 | B | | | | | CAP | | | | | | | | | | | | | | |
| | | F | 60 | 95 | 168 | D | | | | 41.6 | | 5.09 | | | | | | | | |
| | C | | | | | | | | | 61.2 | | 7.9 | | | | | | | | |
| | A | | | | | | 1310 | | 0.05 | | | 10.2 | 1.2 | | | 10. | 30. | | 1.9 | 0.5 |
| | | | | | | | 0 | 0 | 7 | 53.7 | 5.9 | 9 | 4 | 1.83 | 1.01 | 1 | 1 | 2.84 | 7 | 9 |
| P27 | B | | | | | CAP | | | | | | | | | | | | | | |
| | | M | 49 | 72 | 178 | D | | | | 33.6 | | 6.55 | | | | | | | | |
| | C | | | | | | | | | 50.6 | | 10.7 | | | | | | | | |
| | A | | | | | | | | | | | | 0.3 | | | | 28. | | 3.1 | |
| | | | | | | | – | 0 | – | – | 2 | – | 8 | – | – | 9 | 0 | 4.95 | 3 | 1.2 |

| | | | | | | | | | | | | | | | | | | | | |
|---|---|---|---|---|---|---|---|---|---|---|---|---|---|---|---|---|---|---|---|---|
| P28 | B | F | 64 | 89 | 164 | APD | | | | 27.2 | | 5.22 | | | | | | | | |
| | C | | | | | | | | | 38.8 | | 7.52 | | | | | | | | |
| | A | | | | | | 1580 | 150 | 0.05 | | | | | | | 11. | 34. | | 8.2 | 1.9 |
| | | | | | | | 0 | 0 | 4 | 33.4 | 8.3 | 4.04 | 1 | 2.14 | 0.84 | 7 | 9 | 11.3 | 9 | 5 |
| P29 | B | | | | | CAP | | | | | | | | | | | | | | |
| | | M | 31 | 94.3 | 188 | D | | | | 35.3 | | 4.4 | | | | | | | | |
| | C | | | | | | | | | 40 | | 5.56 | | | | | | | | |
| | A | | | | | | | | | | | | 2.1 | | | | 28. | | | 1.6 |
| | | | | | | | 9200 | 200 | 0.76 | 32.6 | 13.5 | – | 7 | 1.58 | 0.71 | 9.3 | 8 | 8.28 | 5.2 | 6 |
| P30 | B | M | 61 | 69 | 175 | APD | | | | 33.1 | | 5.43 | | | | | | | | |
| | C | | | | | | | | | 38.8 | | 6.89 | | | | | | | | |
| | A | | | | | | | | | | | | 0.2 | | | | 36. | | | 0.9 |
| | | | | | | | – | – | – | – | 2.7 | | 1 | – | – | 12 | 9 | 5.73 | 4.2 | 4 |
| P31 | B | F | 51 | 62 | 167 | APD | | | | 24.9 | | 2.29 | | | | | | | | |
| | C | | | | | | | | | 35.4 | | 3.19 | | | | | | | | |
| | A | | | | | | | 250 | | | | | 0.1 | | | 11. | 36. | | | |
| | | | | | | | 7000 | 0 | 0.93 | 41.9 | 3.3 | 1.66 | 3 | 3.43 | 1.2 | 8 | 1 | 7.8 | 4.6 | 2.1 |
| P32 | B | | | | | CAP | | | | | | | | | | | | | | |
| | | F | 71 | 73 | 152 | D | | | | 24 | | 0.8 | | | | | | | | |
| | C | | | | | | | | | 36 | | 1.26 | | | | | | | | |
| | A | | | | | | | 150 | 0.07 | | | | 0.4 | | | 11. | 33. | | 5.5 | 2.0 |
| | | | | | | | 9000 | 0 | 9 | 67.7 | 5.1 | 5.9 | 6 | 1.68 | 1.03 | 3 | 8 | 8.7 | 7 | 6 |
| P33 | B | | | | | CAP | | | | | | | | | | | | | | |
| | | M | 39 | 87 | 182 | D | | | | 28.8 | | 2.23 | | | | | | | | |
| | C | | | | | | | | | 59.3 | | 4.93 | | | | | | | | |
| | A | | | | | | 1060 | 125 | | | | | 0.2 | | | 10. | 33. | | | 1.2 |
| | | | | | | | 0 | 0 | 0.03 | 25.4 | 2.6 | 1.93 | 8 | 3.24 | 1.12 | 8 | 1 | 7.27 | 4.6 | 1 |
| P34 | B | F | 44 | 52.9 | 150 | APD | | | | 18.1 | | 1.89 | | | | | | | | |
| | C | | | | | | | | | 31.7 | | 3.21 | | | | | | | | |
| | A | | | | | | | 100 | 0.11 | | | | 0.4 | | | 10. | 31. | | 3.8 | 1.2 |
| | | | | | | | 8600 | 0 | 9 | 70.4 | 3.1 | 9.42 | 2 | 1.78 | 0.98 | 5 | 8 | 6.36 | 8 | 5 |
| P35 | B | | | | | CAP | | | | | | | | | | | | | | |
| | | M | 57 | 94 | 181 | D | | | | 42.1 | | 5.01 | | | | | | | | |
| | C | | | | | | | | | 59.1 | | 7.49 | | | | | | | | |
| | A | | | | | | 1122 | 100 | 0.05 | | | | | | | | 28. | | 2.2 | 1.5 |
| | | | | | | | 0 | 0 | 4 | 36 | 2 | 2.51 | 0.1 | 1.96 | 0.94 | 9.3 | 3 | 4.46 | 2 | 9 |
| P36 | B | M | 65 | 81.4 | 180 | APD | | | | 23.6 | | 1.74 | | | | | | | | |
| | C | | | | | | | | | 47.6 | | 3.48 | | | | | | | | |
| | A | | | | | | 1040 | 250 | 0.04 | | | | | | | 12. | 36. | | 5.4 | 1.6 |
| | | | | | | | 0 | 0 | 6 | 23.6 | 2 | 1.08 | 0.1 | 2.7 | 0.92 | 1 | 2 | 8.46 | 7 | 6 |
| P37 | B | M | – | 104.7 | 180 | APD | | | | – | | 0.88 | | | | | | | | |
| | C | | | | | | | | | – | | 1.65 | | | | | | | | |
| | A | | | | | | | 150 | | | | | 0.2 | | | 10. | 30. | | 4.6 | 0.8 |
| | | | | | | | 8800 | 0 | 0.09 | 46 | 2.5 | 5.14 | 4 | 2.84 | 1.2 | 1 | 7 | 6.31 | 2 | 4 |

| | | | | | | | | | | | | | | | | | | | | |
|---|---|---|---|---|---|---|---|---|---|---|---|---|---|---|---|---|---|---|---|---|
| P38 | B | F | – | 61.4 | 166 | APD | | | | 18.8 | | 2.38 | | | | | | | | |
| | C | | | | | | | | | 37 | | 4.87 | | | | | | | | |
| | A | | | | | | 1100 | | 0.02 | | | | 0.2 | | | | 23. | | 3.7 | 1.0 |
| | | | | | | | 0 | 800 | 8 | 29.6 | 10 | 2.5 | 1 | 2.79 | 1.13 | 7.3 | 4 | 5.41 | 2 | 8 |
| P39 | B | F | 27 | 56.5 | 166 | APD | | | | 25.8 | | 2.87 | | | | | | | | |
| | C | | | | | | | | | 48.3 | | 6.16 | | | | | | | | |
| | A | | | | | | 1100 | 144 | | | | 10.2 | 0.4 | | | | 27. | | 4.6 | 1.6 |
| | | | | | | | 0 | 0 | 0.06 | 91.1 | 3.9 | 7 | 6 | 1.97 | 1.38 | 8.7 | 3 | 7.18 | 4 | 8 |
| P40 | B | | | | | CAP | | | | | | | | | | | | | | |
| | | M | 54 | 84 | 186 | D | | | | 56 | | 5.61 | | | | | | | | |
| | C | | | | | | | | | 86 | | 9.71 | | | | | | | | |
| | A | | | | | | 1210 | | | | | | 0.1 | | | 11. | 34. | | 3.5 | 0.7 |
| | | | | | | | 0 | 900 | 0.27 | 39.2 | 5.3 | 0.83 | 3 | 2.62 | 0.81 | 3 | 4 | 5.37 | 2 | 7 |
| P41 | B | F | 88 | 67 | 158 | APD | | | | 34 | | | | | | | | | | |
| | C | | | | | | | | | 49.2 | | | | | | | | | | |

Abbreviations: Probe A – sample collected after 24–hour of PD; Probe B – sample collected after 2nd hour of PET; Probe C – sample collected after 4th hour of PET, Sex: F – female, M – male, Weight of patient [kg]; Height of patient [cm]; Age of patient [years]; DO type – type of peritoneal dialysis: CAPD – continuous ambulatory peritoneal dialysis, APD – automated peritoneal dialysis; Vd – the volume of dialysis fluid [ml], Vm – urine output [ml/24 h]; Protein D – protein peritoneal loss [g/ 24 h]; BUN D, BUN D0 – blood urea nitrogen after 24–hour of dialysis/ after 4-hour [mg/dl]; K D, K D0 – creatinine level: after 24–hour of dialysis/ after 4-hour [mg/dl]; Kt/V – parameter of dialysis adequacy; nPCR – normalized protein catabolic rate [g/ kg/ 24–hour]; Hb – hemoglobin [g/dl]; Hct – hematocrit [%]; WBC – white blood cells [x$10^9$/l]; Neutr – neutrocytes [x$10^9$/l]; Limf – lymphocytes [x$10^9$/l]

**Table S2.** Recoveries of ThMP, ThDP, PN, PL, and PM added to dialysis effluents. Values are presented for subsequent additions of standard substances.

| ThMP added [ng/ml] | ThMP found* [ng/ml] | Recovery* [%] | ThDP added [ng/ml] | ThDP found* [ng/ml] | Recovery* [%] | PN added [ng/ml] | PN found* [ng/ml] | Recovery* [%] |
|---|---|---|---|---|---|---|---|---|
| 0.0 | 1.52 ± 0.02 | – | 0.0 | 2.19 ± 0.34 | – | 0.0 | 1.54 ± 0.02 | – |
| 10.0 | 9.14 ± 1.12 | 91.42 ± 3.12 | 10.0 | 8.96 ± 0.82 | 89.61 ± 4.07 | 10.0 | 9.12 ± 0.85 | 91.22 ± 4.81 |
| 25.0 | 23.72 ± 2.05 | 94.48 ± 4.06 | 25.0 | 23.57 ± 1.94 | 94.18 ± 2.37 | 20.0 | 19.04 ± 1.33 | 94.86 ± 2.37 |
| 50.0 | 49.31 ± 2.71 | 97.86 ± 3.72 | 50.0 | 48.12 ± 3.71 | 95.96 ± 4.19 | 25.0 | 23.73 ± 3.19 | 94.12 ± 4.61 |
| **Average recovery [%]** | | 94.68 ± 5.03 | **Average recovery [%]** | | 93.05 ± 4.88 | **Average recovery [%]** | | 93.24 ± 4.17 |
| **PL added [ng/ml]** | **PL found* [ng/ml]** | **Recovery* [%]** | **PM added [ng/ml]** | **PM found* [ng/ml]** | **Recovery* [%]** | | | |
| 0.0 | 5.83 ± 1.06 | – | 0.0 | 1.59 ± 0.26 | – | | | |
| 10.0 | 8.96 ± 0.72 | 89.64 ± 3.28 | 10.0 | 8.77 ± 1.38 | 87.76 ± 4.71 | | | |
| 20.0 | 18.72 ± 2.19 | 93.06 ± 4.03 | 20.0 | 18.94 ± 2.27 | 93.97 ± 3.92 | | | |
| 25.0 | 23.72 ± 3.47 | 93.88 ± 5.19 | 25.0 | 23.61 ± 4.07 | 94.84 ± 5.14 | | | |
| **Average recovery [%]** | | 92.29 ± 5.44 | **Average recovery [%]** | | 92.12 ± 5.37 | | | |

*Average values for n = 5

**Table S3.** Levels of the thiamine monophosphate (ThMP, in ng/mL) and thiamine diphosphate (ThDP, in ng/mL) in dialysis fluids were assessed using the proposed RP-HPLC/FL assay.

| Patient No. | Probe | ThMP* | SD | CV [%] | ThDP* | SD | CV [%] |
|---|---|---|---|---|---|---|---|
| | A | 1.52 | 0.02 | 1.17 | 30.14 | 5.99 | 3.92 |
| P1 | B | – | – | – | 7.05 | 1.74 | 4.88 |
| | C | 0.77 | 0.01 | 2.94 | 23.14 | 5.90 | 5.03 |
| | A | 3.05 | 0.27 | 1.83 | 28.84 | 4.14 | 2.83 |
| P2 | B | – | – | – | 5.45 | 0.15 | 0.53 |
| | C | 0.43 | 0.01 | 0.89 | 20.96 | 2.91 | 2.74 |
| | A | 4.13 | 0.27 | 1.83 | 33.78 | 3.00 | 1.75 |
| P3 | B | – | – | – | 13.02 | 2.02 | 3.06 |
| | C | 1.31 | 0.01 | 0.73 | 33.58 | 3.10 | 1.82 |
| | A | 1.83 | 0.35 | 4.18 | 30.88 | 3.65 | 2.33 |
| P4 | B | – | – | – | 12.96 | 2.71 | 4.13 |
| | C | 0.53 | 0.01 | 1.82 | 15.12 | 2.68 | 3.49 |
| | A | 2.79 | 0.37 | 0.98 | 34.47 | 5.45 | 3.12 |
| P5 | B | – | – | – | 15.55 | 1.44 | 1.83 |
| | C | 0.72 | 0.01 | 1.72 | 22.07 | 0.38 | 0.34 |
| | A | 6.88 | 0.57 | 3.83 | 33.44 | 5.14 | 3.03 |
| P6 | B | 0.81 | 0.21 | 4.82 | 3.98 | 0.56 | 2.79 |
| | C | 1.59 | 0.02 | 3.27 | 32.08 | 5.14 | 3.16 |
| | A | 4.54 | 0.48 | 4.02 | 40.17 | 1.49 | 0.73 |
| P7 | B | – | – | – | 15.67 | 0.55 | 0.69 |
| | C | 0.46 | 0.02 | 3.82 | 35.65 | 1.48 | 0.82 |
| | A | 3.04 | 0.27 | 2.18 | 35.11 | 1.07 | 0.60 |
| P8 | B | – | – | – | 6.44 | 0.15 | 0.45 |
| | C | 0.82 | 0.03 | 5.07 | 14.08 | 0.17 | 0.24 |
| | A | – | – | – | 11.64 | 2.73 | 4.63 |
| P9 | B | – | – | – | 2.19 | 0.34 | 3.04 |
| | C | – | – | – | 9.36 | 0.45 | 0.94 |
| | A | 4.58 | 0.38 | 4.12 | 26.16 | 1.09 | 0.82 |
| P10 | B | – | – | – | 8.08 | 1.06 | 2.58 |
| | C | 1.40 | 0.27 | 3.28 | 15.44 | 1.20 | 1.53 |
| | A | 1.39 | 0.31 | 2.83 | 50.24 | 2.52 | 0.99 |
| P11 | B | – | – | – | 31.16 | 0.73 | 0.46 |
| | C | 0.85 | 0.04 | 2.73 | 40.14 | 8.14 | 4.00 |
| | A | – | – | – | 8.84 | 1.80 | 4.02 |
| P12 | B | – | – | – | 5.45 | 0.37 | 1.33 |
| | C | – | – | – | 10.96 | 0.18 | 0.33 |
| | A | 2.43 | 0.29 | 4.17 | 43.78 | 3.51 | 1.58 |
| P13 | B | – | – | – | 3.02 | 0.69 | 4.52 |
| | C | 0.84 | 0.03 | 3.64 | 33.58 | 8.58 | 5.04 |

| | | | | | | | |
|---|---|---|---|---|---|---|---|
| | A | 3.62 | 0.57 | 4.08 | 20.88 | 4.96 | 4.69 |
| P14 | B | – | – | – | 12.96 | 1.60 | 2.43 |
| | C | 2.24 | 0.28 | 3.64 | 15.12 | 1.10 | 1.44 |
| | A | – | – | – | 14.47 | 0.30 | 0.41 |
| P15 | B | – | – | – | 5.55 | 1.02 | 3.62 |
| | C | – | – | – | 12.07 | 0.07 | 0.12 |
| | A | – | – | – | 23.44 | 2.67 | 2.25 |
| P16 | B | – | – | – | 2.98 | 0.10 | 0.63 |
| | C | – | – | – | 12.94 | 0.20 | 0.31 |
| | A | 0.89 | 0.02 | 3.63 | 40.17 | 0.98 | 0.48 |
| P17 | B | – | – | – | 25.67 | 0.78 | 0.60 |
| | C | – | – | – | 35.65 | 7.07 | 3.91 |
| | A | 5.66 | 1.07 | 3.54 | 35.11 | 0.34 | 0.19 |
| P18 | B | – | – | – | 6.44 | 0.29 | 0.90 |
| | C | 1.28 | 0.48 | 5.12 | 10.85 | 0.50 | 0.90 |
| | A | – | – | – | 11.64 | 1.69 | 2.87 |
| P19 | B | – | – | – | 2.19 | 0.63 | 5.66 |
| | C | – | – | – | 9.36 | 0.50 | 1.05 |
| | A | – | – | – | 26.16 | 1.71 | 1.29 |
| P20 | B | – | – | – | 8.08 | 0.50 | 1.22 |
| | C | – | – | – | 25.44 | 2.77 | 2.15 |
| | A | 1.25 | 0.37 | 4.92 | 30.24 | 4.37 | 2.85 |
| P21 | B | – | – | – | 1.16 | 0.18 | 3.08 |
| | C | – | – | – | 12.04 | 2.39 | 3.91 |
| | A | – | – | – | 7.35 | 1.54 | 4.12 |
| P22 | B | – | – | – | 1.22 | 0.08 | 1.26 |
| | C | – | – | – | 3.18 | 0.11 | 0.70 |
| | A | 3.27 | 1.05 | 5.63 | 41.17 | 4.55 | 2.18 |
| P23 | B | 0.53 | 0.02 | 0.13 | 10.13 | 0.06 | 0.11 |
| | C | 0.91 | 0.26 | 3.72 | 24.16 | 1.73 | 1.41 |
| | A | – | – | – | 10.67 | 0.23 | 0.43 |
| P24 | B | – | – | – | 1.03 | 0.02 | 0.33 |
| | C | – | – | – | 5.44 | 0.09 | 0.33 |
| | A | 0.57 | 0.03 | 4.04 | 27.95 | 1.30 | 0.92 |
| P25 | B | – | – | – | 3.12 | 0.40 | 2.54 |
| | C | – | – | – | 7.91 | 0.05 | 0.12 |
| | A | 1.29 | 0.59 | 4.52 | 46.12 | 0.72 | 0.31 |
| P26 | B | – | – | – | 2.74 | 0.80 | 5.76 |
| | C | – | – | – | 12.64 | 2.90 | 4.53 |
| | A | 1.23 | 0.37 | 3.16 | 20.05 | 5.35 | 5.26 |
| P27 | B | – | – | – | 3.27 | 0.22 | 1.30 |
| | C | – | – | – | 7.54 | 0.15 | 0.39 |
| | A | 1.79 | 0.47 | 4.72 | 19.77 | 0.99 | 0.99 |
| P28 | B | – | – | – | 4.02 | 0.25 | 1.24 |

| | | | | | | | |
|---|---|---|---|---|---|---|---|
| | C | – | – | – | 11.03 | 0.18 | 0.33 |
| | A | 1.04 | 0.27 | 2.73 | 27.02 | 1.90 | 1.39 |
| P29 | B | – | – | – | 2.65 | 0.24 | 1.76 |
| | C | – | – | – | 14.03 | 0.57 | 0.80 |
| | A | 6.83 | 1.03 | 4.72 | 35.67 | 0.24 | 0.13 |
| P30 | B | 1.66 | 0.37 | 2.95 | 13.02 | 0.69 | 1.04 |
| | C | 3.72 | 0.72 | 4.28 | 27.02 | 1.34 | 0.98 |
| | A | – | – | – | 5.12 | 0.79 | 3.03 |
| P31 | B | – | – | – | 0.76 | 0.26 | 6.83 |
| | C | – | – | – | 2.34 | 0.33 | 2.75 |
| | A | 0.93 | 0.03 | 1.27 | 13.07 | 2.35 | 3.54 |
| P32 | B | – | – | – | 0.45 | 0.02 | 0.90 |
| | C | – | – | – | 3.01 | 0.33 | 2.14 |
| | A | 1.30 | 0.47 | 4.07 | 42.02 | 1.58 | 0.74 |
| P33 | B | – | – | – | 1.04 | 0.05 | 0.93 |
| | C | 0.84 | 0.12 | 3.84 | 13.13 | 1.76 | 2.64 |
| | A | – | – | – | 5.73 | 0.43 | 1.48 |
| P34 | B | – | – | – | 0.57 | 0.04 | 1.31 |
| | C | – | – | – | 2.17 | 0.08 | 0.76 |
| | A | 3.21 | 0.75 | 4.82 | 48.04 | 2.05 | 0.84 |
| P35 | B | – | – | – | 15.72 | 1.21 | 1.52 |
| | C | 0.61 | 0.02 | 0.94 | 27.18 | 0.39 | 0.28 |
| | A | 0.53 | 0.02 | 1.73 | 22.04 | 1.48 | 1.32 |
| P36 | B | – | – | – | 1.83 | 0.00 | 0.01 |
| | C | – | – | – | 5.94 | 0.11 | 0.35 |
| | A | 1.67 | 0.47 | 4.27 | 37.12 | 1.17 | 0.62 |
| P37 | B | – | – | – | 2.27 | 0.04 | 0.36 |
| | C | 0.48 | 0.02 | 3.77 | 17.06 | 1.05 | 1.21 |
| | A | – | – | – | 13.64 | 0.03 | 0.04 |
| P38 | B | – | – | – | 3.92 | 0.03 | 0.17 |
| | C | – | – | – | 7.27 | 0.12 | 0.33 |
| | A | 0.97 | 0.03 | 0.83 | 26.08 | 0.65 | 0.49 |
| P39 | B | – | – | – | 3.18 | 0.08 | 0.47 |
| | C | – | – | – | 13.62 | 0.10 | 0.14 |
| | A | 6.57 | 0.94 | 3.72 | 40.28 | 0.04 | 0.02 |
| P40 | B | – | – | – | 5.06 | 0.03 | 0.10 |
| | C | 2.01 | 0.57 | 5.01 | 22.67 | 3.10 | 2.70 |
| | A | 0.63 | 0.02 | 1.79 | 16.37 | 5.61 | 6.76 |
| P41 | B | – | – | – | 3.28 | 1.09 | 6.58 |
| | C | – | – | – | 6.82 | 0.36 | 1.03 |

Abbreviations: $^{*}$average for n=3; (–) analyte not detected or value below LOQ; SD standard deviation, CI (confidence interval) 95%; CV coefficient of variation; Probe A – samples collected after 24 hours of dialysis; Probe B – samples collected after 2$^{nd}$ hour of PET test; Probe C – samples collected after 4$^{th}$ hour of PET test

**Table S4.** Levels of the pyridoxine (PN, in ng/mL), pyridoxal (PL, in ng/mL) and pyridoxamine (PM, in ng/mL) in dialysis fluids assessed using proposed RP-HPLC/FL assay.

| Patient No. | Probe | PN* | SD | CV [%] | PL* | SD | CV [%] | PM* | SD | CV [%] |
|---|---|---|---|---|---|---|---|---|---|---|
| P1 | A | 1.72 | 0.03 | 0.35 | 6.71 | 1.42 | 4.18 | 1.26 | 0.01 | 0.23 |
| | B | 1.06 | 0.17 | 3.16 | 5.72 | 0.16 | 0.56 | 0.08 | 0.01 | 0.15 |
| | C | 1.31 | 0.14 | 2.14 | 5.18 | 0.64 | 2.45 | 0.51 | 0.05 | 2.12 |
| P2 | A | 1.54 | 0.02 | 0.25 | 5.83 | 1.06 | 3.58 | 1.59 | 0.26 | 3.21 |
| | B | 0.08 | 0.00 | 0.02 | 4.15 | 0.73 | 3.47 | 0.27 | 0.03 | 2.02 |
| | C | 1.15 | 0.18 | 3.09 | 4.82 | 0.53 | 2.15 | 0.50 | 0.01 | 0.02 |
| P3 | A | 1.18 | 0.19 | 3.17 | 12.41 | 2.59 | 4.12 | 1.37 | 0.01 | 0.15 |
| | B | 0.38 | 0.01 | 0.65 | 7.55 | 1.94 | 5.07 | 0.22 | 0.04 | 3.17 |
| | C | 0.75 | 0.13 | 3.48 | 1.69 | 0.39 | 4.56 | 0.44 | 0.01 | 0.25 |
| P4 | A | 1.53 | 0.03 | 0.45 | 17.05 | 3.38 | 3.91 | 2.53 | 0.45 | 3.48 |
| | B | 0.49 | 0.02 | 0.94 | 7.03 | 1.34 | 3.76 | 0.62 | 0.01 | 0.05 |
| | C | 0.75 | 0.04 | 1.18 | 16.02 | 1.75 | 2.15 | 2.11 | 0.19 | 1.76 |
| P5 | A | 1.71 | 0.15 | 1.71 | 16.23 | 1.38 | 1.68 | 0.62 | 0.02 | 0.54 |
| | B | 0.73 | 0.06 | 1.62 | 7.37 | 1.55 | 4.15 | 0.22 | 0.01 | 0.68 |
| | C | 0.86 | 0.04 | 0.92 | 15.32 | 2.47 | 3.18 | 0.46 | 0.02 | 0.95 |
| P6 | A | 2.57 | 0.11 | 0.84 | 17.13 | 3.01 | 3.47 | 1.37 | 0.22 | 3.15 |
| | B | 0.15 | 0.01 | 1.54 | 16.63 | 2.32 | 2.75 | 0.37 | 0.01 | 0.09 |
| | C | 0.53 | 0.07 | 2.65 | 16.20 | 1.37 | 1.67 | 0.80 | 0.02 | 0.48 |
| P7 | A | 0.83 | 0.05 | 1.25 | 7.37 | 0.63 | 1.69 | 2.06 | 0.07 | 0.68 |
| | B | 0.09 | 0.01 | 3.14 | 7.19 | 0.96 | 2.64 | 0.19 | 0.01 | 0.35 |
| | C | 0.36 | 0.01 | 0.34 | 7.08 | 1.14 | 3.18 | 2.41 | 0.51 | 4.19 |
| P8 | A | 1.00 | 0.09 | 1.74 | 8.13 | 1.02 | 2.48 | 3.58 | 0.10 | 0.54 |
| | B | 0.68 | 0.03 | 0.95 | 4.99 | 0.93 | 3.67 | 1.26 | 0.04 | 0.68 |
| | C | 0.77 | 0.11 | 2.74 | 5.22 | 0.84 | 3.18 | 2.01 | 0.05 | 0.48 |
| P9 | A | 1.71 | 0.06 | 0.64 | 7.35 | 0.92 | 2.48 | 2.99 | 0.06 | 0.39 |
| | B | 0.31 | 0.06 | 3.74 | 2.14 | 0.32 | 2.97 | 0.39 | 0.01 | 0.48 |
| | C | 0.81 | 0.08 | 1.84 | 6.20 | 0.98 | 3.12 | 0.42 | 0.01 | 0.64 |
| P10 | A | 2.03 | 0.20 | 1.95 | 7.73 | 1.50 | 3.84 | 0.75 | 0.01 | 0.37 |
| | B | 1.38 | 0.26 | 3.71 | 6.31 | 1.51 | 4.71 | 0.12 | 0.00 | 0.08 |
| | C | 1.58 | 0.07 | 0.91 | 6.83 | 1.27 | 3.68 | 0.68 | 0.03 | 0.84 |
| P11 | A | 0.65 | 0.02 | 0.67 | 6.62 | 0.83 | 2.48 | 0.78 | 0.04 | 1.12 |
| | B | 0.12 | 0.02 | 3.49 | 6.18 | 0.31 | 0.99 | 0.24 | 0.01 | 0.64 |
| | C | 0.30 | 0.03 | 2.14 | 6.88 | 0.99 | 2.84 | 0.81 | 0.06 | 1.36 |
| P12 | A | 1.64 | 0.08 | 0.94 | 7.58 | 1.12 | 2.91 | 0.59 | 0.01 | 0.08 |
| | B | 0.68 | 0.06 | 1.64 | 6.96 | 1.12 | 3.18 | 0.25 | 0.01 | 0.98 |
| | C | 1.15 | 0.07 | 1.28 | 7.16 | 1.47 | 4.05 | 0.68 | 0.02 | 0.45 |
| P13 | A | 2.61 | 0.33 | 2.47 | 7.13 | 1.49 | 4.12 | 0.88 | 0.03 | 0.74 |
| | B | 0.98 | 0.05 | 0.94 | 3.63 | 0.67 | 3.64 | 0.12 | 0.01 | 0.64 |
| | C | 1.35 | 0.11 | 1.64 | 5.90 | 0.95 | 3.19 | 0.72 | 0.01 | 0.05 |

| | | | | | | | | | | |
|---|---|---|---|---|---|---|---|---|---|---|
| | A | 1.43 | 0.18 | 2.49 | 8.04 | 1.17 | 2.87 | 1.64 | 0.07 | 0.82 |
| P14 | B | 0.00 | 0.00 | 0.00 | 2.39 | 0.24 | 1.95 | 0.68 | 0.01 | 0.03 |
| | C | 0.80 | 0.10 | 2.54 | 7.77 | 0.65 | 1.64 | 1.37 | 0.05 | 0.74 |
| | A | 2.13 | 0.16 | 1.47 | 9.79 | 0.68 | 1.38 | 1.91 | 0.07 | 0.76 |
| P15 | B | 1.01 | 0.04 | 0.84 | 4.06 | 0.79 | 3.86 | 1.01 | 0.04 | 0.84 |
| | C | 1.81 | 0.06 | 0.64 | 6.42 | 1.04 | 3.19 | 1.66 | 0.13 | 1.54 |
| | A | 2.25 | 0.09 | 0.83 | 7.67 | 1.10 | 2.84 | 1.27 | 0.05 | 0.78 |
| P16 | B | 0.00 | 0.00 | 0.00 | 2.27 | 0.31 | 2.68 | 0.72 | 0.01 | 0.24 |
| | C | 1.19 | 0.15 | 2.41 | 5.04 | 0.55 | 2.14 | 0.88 | 0.07 | 1.65 |
| | A | 1.61 | 0.05 | 0.67 | 7.93 | 1.48 | 3.68 | 2.93 | 0.17 | 1.12 |
| P17 | B | 0.31 | 0.03 | 1.64 | 0.48 | 0.08 | 3.19 | 0.88 | 0.04 | 0.84 |
| | C | 0.95 | 0.08 | 1.75 | 3.16 | 0.58 | 3.65 | 1.40 | 0.10 | 1.37 |
| | A | 2.11 | 0.14 | 1.34 | 7.90 | 0.23 | 0.58 | 1.62 | 0.01 | 0.05 |
| P18 | B | 1.03 | 0.09 | 1.64 | 0.87 | 0.06 | 1.38 | 1.14 | 0.18 | 3.14 |
| | C | 1.35 | 0.13 | 1.95 | 2.96 | 0.40 | 2.68 | 1.27 | 0.12 | 1.84 |
| | A | 1.59 | 0.13 | 1.62 | 8.44 | 0.92 | 2.15 | 1.50 | 0.04 | 0.51 |
| P19 | B | 1.09 | 0.14 | 2.48 | 0.09 | 0.02 | 3.48 | 1.33 | 0.23 | 3.47 |
| | C | 1.17 | 0.16 | 2.68 | 4.50 | 0.50 | 2.17 | 1.97 | 0.41 | 4.12 |
| | A | 1.45 | 0.05 | 0.64 | 8.38 | 1.14 | 2.68 | 2.17 | 0.30 | 2.74 |
| P20 | B | 0.68 | 0.06 | 1.61 | 0.89 | 0.06 | 1.39 | 0.46 | 0.01 | 0.08 |
| | C | 0.65 | 0.03 | 0.94 | 4.94 | 0.95 | 3.78 | 0.58 | 0.08 | 2.85 |
| | A | 0.93 | 0.03 | 0.64 | 8.13 | 1.52 | 3.68 | 1.93 | 0.16 | 1.67 |
| P21 | B | 0.81 | 0.06 | 1.35 | 1.96 | 0.32 | 3.17 | 0.87 | 0.06 | 1.31 |
| | C | 0.72 | 0.05 | 1.36 | 2.53 | 0.53 | 4.12 | 0.96 | 0.10 | 2.15 |
| | A | 1.91 | 0.05 | 0.47 | 8.53 | 1.38 | 3.18 | 1.48 | 0.28 | 3.73 |
| P22 | B | 1.39 | 0.12 | 1.64 | 3.08 | 0.33 | 2.09 | 0.40 | 0.06 | 3.17 |
| | C | 0.96 | 0.03 | 0.67 | 6.60 | 0.34 | 1.02 | 0.92 | 0.10 | 2.09 |
| | A | 2.12 | 0.34 | 3.18 | 8.37 | 0.28 | 0.67 | 0.99 | 0.14 | 2.71 |
| P23 | B | 1.31 | 0.16 | 2.48 | 1.18 | 0.17 | 2.81 | 0.76 | 0.08 | 2.14 |
| | C | 1.69 | 0.23 | 2.68 | 3.20 | 0.42 | 2.61 | 0.23 | 0.04 | 3.08 |
| | A | 1.81 | 0.15 | 1.64 | 8.23 | 1.31 | 3.14 | 0.96 | 0.15 | 3.18 |
| P24 | B | 1.17 | 0.15 | 2.45 | 0.22 | 0.05 | 4.12 | 0.56 | 0.09 | 3.33 |
| | C | 1.07 | 0.15 | 2.68 | 1.95 | 0.09 | 0.88 | 0.64 | 0.10 | 3.08 |
| | A | 1.56 | 0.07 | 0.84 | 8.07 | 0.89 | 2.17 | 2.73 | 0.28 | 2.05 |
| P25 | B | 0.70 | 0.03 | 0.92 | 0.70 | 0.13 | 3.71 | 0.69 | 0.07 | 1.97 |
| | C | 0.88 | 0.07 | 1.64 | 3.31 | 0.52 | 3.08 | 1.20 | 0.05 | 0.88 |
| | A | 1.43 | 0.09 | 1.25 | 9.16 | 1.37 | 2.94 | 1.32 | 0.06 | 0.91 |
| P26 | B | 0.66 | 0.09 | 2.71 | 1.49 | 0.19 | 2.48 | 1.19 | 0.07 | 1.08 |
| | C | 0.86 | 0.18 | 4.12 | 3.63 | 0.58 | 3.17 | 1.64 | 0.15 | 1.82 |
| | A | 1.30 | 0.25 | 3.75 | 11.26 | 2.29 | 4.02 | 2.69 | 0.09 | 0.68 |
| P27 | B | 0.51 | 0.11 | 4.31 | 6.57 | 1.30 | 3.91 | 0.36 | 0.04 | 2.14 |
| | C | 1.41 | 0.23 | 3.17 | 8.25 | 1.87 | 4.48 | 1.89 | 0.09 | 0.91 |
| | A | 0.69 | 0.06 | 1.82 | 5.84 | 0.65 | 2.18 | 2.05 | 0.28 | 2.68 |
| P28 | B | 0.39 | 0.01 | 0.66 | 1.76 | 0.29 | 3.26 | 0.14 | 0.02 | 2.18 |

| | | | | | | | | | | |
|---|---|---|---|---|---|---|---|---|---|---|
| | C | 0.58 | 0.03 | 0.98 | 2.35 | 0.26 | 2.18 | 1.14 | 0.18 | 3.04 |
| | A | 1.49 | 0.09 | 1.22 | 12.06 | 2.45 | 4.01 | 1.82 | 0.26 | 2.78 |
| P29 | B | 0.86 | 0.04 | 0.98 | 2.98 | 0.62 | 4.08 | 0.80 | 0.07 | 1.69 |
| | C | 0.97 | 0.07 | 1.38 | 9.45 | 1.76 | 3.67 | 0.84 | 0.05 | 1.06 |
| | A | 0.89 | 0.11 | 2.47 | 17.26 | 2.75 | 3.14 | 1.09 | 0.21 | 3.74 |
| P30 | B | 0.59 | 0.06 | 2.08 | 2.06 | 0.30 | 2.88 | 1.04 | 0.14 | 2.68 |
| | C | 0.62 | 0.07 | 2.11 | 7.94 | 1.07 | 2.67 | 0.81 | 0.07 | 1.59 |
| | A | 1.55 | 0.07 | 0.94 | 13.38 | 2.36 | 3.48 | 2.08 | 0.21 | 1.97 |
| P31 | B | 0.98 | 0.08 | 1.68 | 3.82 | 0.36 | 1.88 | 0.43 | 0.07 | 3.08 |
| | C | 0.96 | 0.13 | 2.66 | 5.33 | 0.99 | 3.67 | 1.07 | 0.11 | 2.09 |
| | A | 1.99 | 0.25 | 2.47 | 15.27 | 2.74 | 3.54 | 1.67 | 0.34 | 4.08 |
| P32 | B | 1.30 | 0.20 | 3.09 | 0.75 | 0.15 | 4.00 | 0.46 | 0.02 | 0.73 |
| | C | 1.34 | 0.21 | 3.14 | 6.07 | 0.98 | 3.19 | 1.02 | 0.06 | 1.12 |
| | A | 1.54 | 0.19 | 2.47 | 11.48 | 2.42 | 4.15 | 1.68 | 0.18 | 2.08 |
| P33 | B | 1.48 | 0.07 | 0.99 | 2.42 | 0.26 | 2.08 | 0.18 | 0.02 | 2.71 |
| | C | 0.84 | 0.06 | 1.44 | 4.21 | 0.35 | 1.64 | 1.01 | 0.03 | 0.64 |
| | A | 0.91 | 0.08 | 1.68 | 10.02 | 0.56 | 1.11 | 0.46 | 0.03 | 1.17 |
| P34 | B | 0.85 | 0.16 | 3.74 | 0.17 | 0.03 | 3.25 | – | – | 0.94 |
| | C | 0.98 | 0.13 | 2.67 | 1.54 | 0.22 | 2.88 | 0.37 | 0.04 | 2.15 |
| | A | 1.06 | 0.09 | 1.74 | 8.37 | 1.33 | 3.14 | 1.70 | 0.22 | 2.61 |
| P35 | B | 1.03 | 0.10 | 1.98 | 0.47 | 0.05 | 2.19 | 1.09 | 0.19 | 3.42 |
| | C | 1.04 | 0.14 | 2.67 | 4.40 | 0.19 | 0.87 | 0.72 | 0.02 | 0.67 |
| | A | 1.29 | 0.16 | 2.48 | 8.61 | 1.38 | 3.17 | 0.75 | 0.06 | 1.64 |
| P36 | B | 0.86 | 0.12 | 2.67 | 0.82 | 0.18 | 4.41 | 0.08 | 0.01 | 0.96 |
| | C | 1.06 | 0.09 | 1.61 | 3.22 | 0.62 | 3.82 | 0.91 | 0.05 | 1.16 |
| | A | 1.15 | 0.10 | 1.77 | 9.58 | 1.30 | 2.67 | 2.28 | 0.36 | 3.12 |
| P37 | B | 1.04 | 0.20 | 3.82 | 1.08 | 0.09 | 1.61 | 0.85 | 0.16 | 3.76 |
| | C | 0.93 | 0.17 | 3.64 | 1.55 | 0.24 | 3.10 | 2.24 | 0.07 | 0.58 |
| | A | 1.88 | 0.23 | 2.42 | 11.75 | 1.32 | 2.22 | 2.89 | 0.25 | 1.69 |
| P38 | B | 1.56 | 0.15 | 1.88 | 4.99 | 0.42 | 1.67 | 0.39 | 0.06 | 3.08 |
| | C | 0.84 | 0.05 | 1.09 | 7.66 | 0.26 | 0.67 | 1.11 | 0.08 | 1.51 |
| | A | 1.47 | 0.15 | 2.07 | 7.78 | 0.65 | 1.64 | 3.64 | 0.58 | 3.14 |
| P39 | B | 0.92 | 0.08 | 1.77 | 0.23 | 0.04 | 3.57 | 0.78 | 0.02 | 0.48 |
| | C | 1.06 | 0.05 | 0.94 | 2.25 | 0.51 | 4.49 | 2.67 | 0.65 | 4.78 |
| | A | 1.60 | 0.17 | 2.14 | 7.85 | 1.12 | 2.81 | 3.35 | 0.02 | 0.12 |
| P40 | B | 1.09 | 0.17 | 3.11 | 0.46 | 0.04 | 1.67 | 1.07 | 0.11 | 2.07 |
| | C | 1.26 | 0.26 | 4.06 | 3.66 | 0.09 | 0.49 | 2.37 | 0.11 | 0.92 |
| | A | 1.41 | 0.05 | 0.67 | 9.22 | 1.42 | 3.04 | 3.22 | 0.12 | 0.74 |
| P41 | B | 1.08 | 0.14 | 2.48 | 1.52 | 0.16 | 2.05 | 1.01 | 0.08 | 1.49 |
| | C | 1.34 | 0.12 | 1.83 | 9.03 | 1.26 | 2.76 | 2.18 | 0.45 | 4.07 |

Abbreviations: *Average for n=3; (–) analyte not detected or value below LOQ; SD standard deviation, CI (confidence interval) 95%; CV coefficient of variation; Probe A – samples collected after 24 hours of dialysis; Probe B – samples collected after 2nd hour of PET test; Probe C – samples collected after 4th hour of PET test
